# Hydrogenated vacancies lock dislocations in aluminium

**DOI:** 10.1038/ncomms13341

**Published:** 2016-11-03

**Authors:** Degang Xie, Suzhi Li, Meng Li, Zhangjie Wang, Peter Gumbsch, Jun Sun, Evan Ma, Ju Li, Zhiwei Shan

**Affiliations:** 1 Center for Advancing Materials Performance from the Nanoscale (CAMP-Nano) & Hysitron Applied Research Center in China (HARCC), State Key Laboratory for Mechanical Behavior of Materials, Xi'an Jiaotong University, Xi′an 710049, China; 2 Institute for Applied Materials, Karlsruhe Institute of Technology, 76131 Karlsruhe, Germany; 3 Fraunhofer-Institut für Werkstoffmechanik IWM, 79108 Freiburg, Germany; 4 Department of Materials Science and Engineering, Johns Hopkins University, Baltimore, Maryland 21218, USA; 5 Department of Nuclear Science and Engineering and Department of Materials Science and Engineering, Massachusetts Institute of Technology, Cambridge, Massachusetts 02139, USA

## Abstract

Due to its high diffusivity, hydrogen is often considered a weak inhibitor or even a promoter of dislocation movements in metals and alloys. By quantitative mechanical tests in an environmental transmission electron microscope, here we demonstrate that after exposing aluminium to hydrogen, mobile dislocations can lose mobility, with activating stress more than doubled. On degassing, the locked dislocations can be reactivated under cyclic loading to move in a stick-slip manner. However, relocking the dislocations thereafter requires a surprisingly long waiting time of ∼10^3^ s, much longer than that expected from hydrogen interstitial diffusion. Both the observed slow relocking and strong locking strength can be attributed to superabundant hydrogenated vacancies, verified by our atomistic calculations. Vacancies therefore could be a key plastic flow localization agent as well as damage agent in hydrogen environment.

## Abstract (web summary)

Due to its high diffusivity, hydrogen is considered a weak inhibitor or even a promoter of dislocation movements in metals and alloys. Here the authors quantitatively demonstrate that after exposing aluminium to hydrogen, mobile dislocations can lose mobility, due to segregation of hydrogenated vacancies to dislocations.

Hydrogen is incorporated into many industrially important metals during processing or service, and often has deleterious consequence on mechanical properties that is commonly referred to as hydrogen embrittlement (HE). Despite numerous efforts over the past century^1^, the exact mechanism of hydrogen effects on the ability of the material to plastically deform remains controversial. For example, both softening and hardening effects were observed in macroscopic tensile tests^2^,^3^,^4^,^5^. Also, the fracture surfaces of hydrogen-embrittled materials usually contain features indicative of pronounced local plastic deformation^6^,^7^,^8^. Knowing exactly how hydrogen interacts with dislocations—the primary carriers of plasticity, is therefore important.

There are debates over hydrogen effects on dislocation motion. According to Cottrell's theory, diffusible atoms are expected to cause a resistance to dislocation motion by forming an atmosphere around the dislocation, that could drag it^9^. Birnbaum and Robertson *et al*.^10^,^11^ proposed that by reducing the long-range elastic interaction between dislocations, hydrogen generates a ‘shielding' effect that makes individual dislocation motion easier in a train of same-sign dislocations. However, their experimental set-up may not guarantee that the applied ‘constant strain' or ‘constant stress' was truly constant when tens of torrs of hydrogen gas flooded the TEM chamber, as fresh dislocations were seen to be generated in the ‘constant strain' stage^12^. Song and Curtin^13^,^14^ stated based on atomistic calculations that the hydrogen interstitial atmosphere dressing a dislocation provides little shielding of elastic interaction; it also reduces rather than enhances the dislocation mobility. These mechanisms are hydrogen concentration, temperature and strain-rate dependent, and are not necessarily mutually exclusive.

Hydrogen has been experimentally found to facilitate the preservation of superabundant vacancies (SAVs) in a large variety of metals and alloys^15^,^16^,^17^. With SAVs inside, a dislocation can meet and even pick up these tiny ‘obstacles' along its gliding path^18^, or attract SAVs toward it during waiting time. The behaviour of dislocations is thus expected to be modified in the presence of hydrogen-stabilized SAVs. In some measurements^16^, the vacancy concentration can even exceed the hydrogen concentration. The role of SAVs in dislocation motion was however not taken into account in previous studies of hydrogen–dislocation interaction, to the best of our knowledge. The SAVs were also supposed to play a role in the fracture of metals^18^,^19^,^20^,^21^,^22^,^23^,^24^, as a form of microscale damage (the smallest void or bubble) that reduces the total metal–metal coordination number in a sample.

Since dislocation dynamics can be influenced by both hydrogen interstitial and hydrogenated vacancy, and neither of them can be directly visualized, experimental verification of hydrogen/vacancy effect on dislocation behaviour is very challenging. To resolve this issue, we note that relative to the fast interstitial diffusion of hydrogen atoms, the diffusion of hydrogenated vacancy would be at least two orders of magnitude slower at room temperature^16^,^25^,^26^. Therefore, an effective way to differentiate the two is to measure the time scale of their trapping kinetics on individual dislocation motion through cyclic loading, with different in-between aging times, in vacuum and in hydrogen environment, respectively. We show that the segregation time for hydrogen to lock dislocation motion is at least two orders more than the prediction based on interstitial hydrogen diffusion. Thus, hydrogen-vacancy complexes are proposed to be the effective diffusing species, at odds with the widely accepted knowledge of hydrogen–dislocation interaction. These results are relevant for modelling hydrogen embrittlement.

## Results

### *In situ* mechanical tests

**Figure 1:**
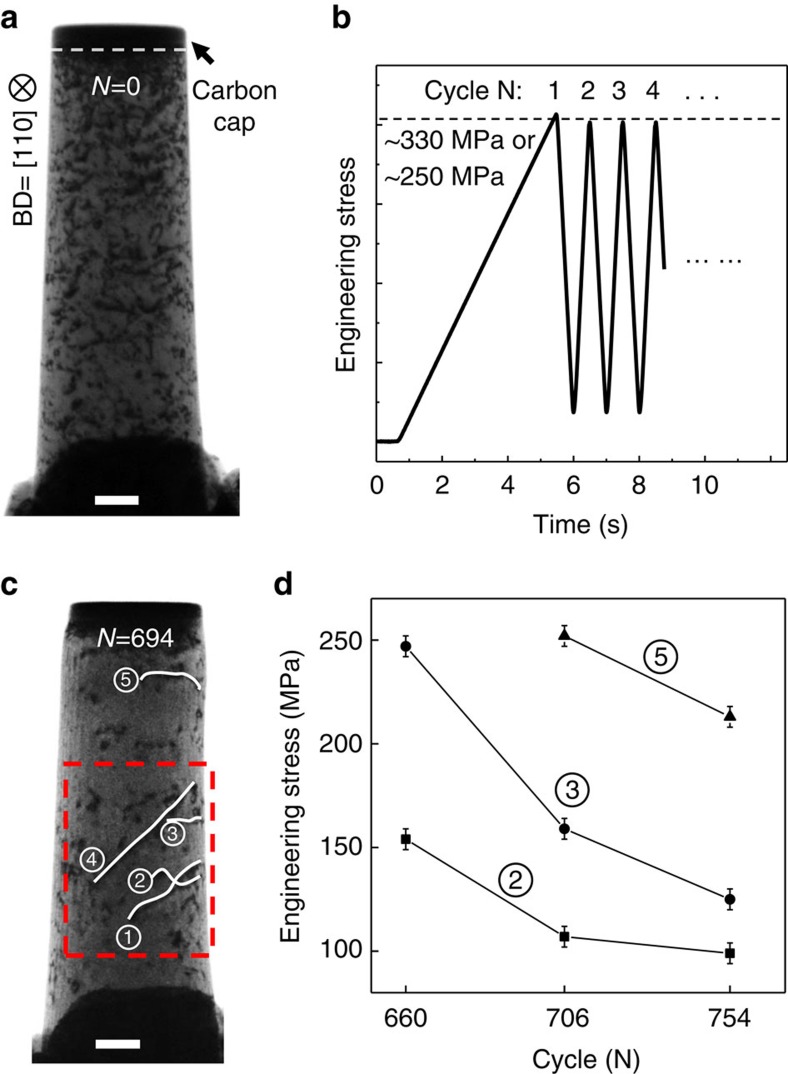
Mechanical annealing and stabilization of dislocations inside the aluminium pillar. (**a**) The as-fabricated pillar shows high dislocation density, and a carbon cap is deposited atop the pillar as a buffer layer to ensure stable contact between multiple tests. (**b**) The loading curve used in cyclic compression tests. (**c**) After 694 cycles of cyclic compression, the dislocation density is dramatically decreased, leaving only 5 mobile dislocations. The region in the red dashed rectangle is examined in detail, see Figs 2, 3, 4. (**d**) The measured critical stress for dislocation forward-jump decreases with loading cycle, for each dislocation. Error bars represent standard deviation. All scale bars are 200 nm.

A single-crystal aluminium pillar sample with diameter of ∼620 nm is chosen because dislocations can be imaged easily and clearly inside TEM. The pillar top is covered by a deposited carbonaceous layer of ∼100 nm to buffer disturbances from rough contacts between the Al pillar and the diamond punch during multiple compression tests. As shown in Fig. 1a, the as-prepared pillar has a high density of dislocations. Therefore, we take advantage of the ‘cyclic healing' phenomenon^27^,^28^ to clean up the interior of the pillar, using the loading function shown in Fig. 1b with peak stresses of ∼330 or ∼250 MPa. The detailed healing process is in Supplementary Fig. 1 and Supplementary Note 1. Shown in Fig. 1c are samples after the cyclic healing, in which only a few mobile dislocations delineated by the white solid lines remained. It is interesting that three of the five dislocations marked in Fig. 1c exhibited sudden forward jump at a critical threshold stress. Presumably, this should arise from overcoming some pinning points along the dislocation line. The slow loading rate in the leading cycle of each cyclic test group allows us to resolve the de-pinning stress of each dislocation by mapping the stress data to the movie frames (Supplementary Fig. 2). As shown in Fig. 1d, the critical stress for these dislocation jumps decreases with increasing cycle number. This indicates that the more loading cycles these dislocations have experienced, the weaker the pinning becomes. For example, from *N*=660 to 754 (*N* indicates number of cycles), the critical stress for the jump of dislocation #2 dropped from ∼150 to ∼100 MPa.

**Figure 2:**
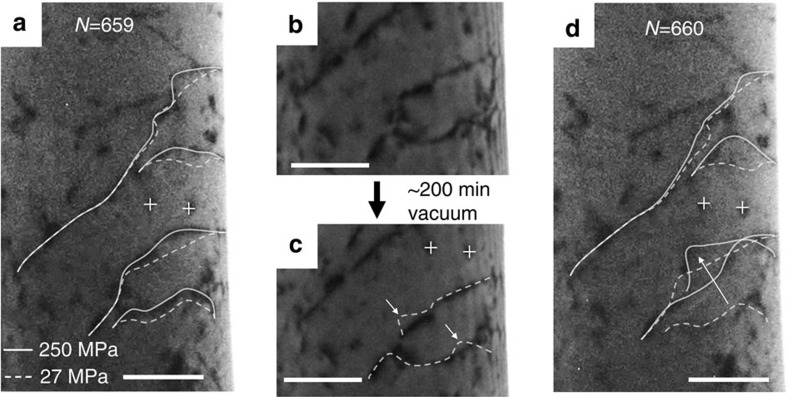
The effect of vacuum aging on dislocation movements in hydrogen-free sample. (**a**) Dislocation motion in the cycle immediately before the vacuum aging. Dashed line and solid line indicate dislocation positions at the valley stress (27 MPa) and peak stress (250 MPa), respectively. (**b**) and (**c**) Dislocation configurations before and after vacuum aging, respectively, during which some of the defects disappeared and the curved dislocation segment tends to be straightened (marked with white arrow). (**d**) After ‘aging' in vacuum for about 200 min at room temperature. All the moving dislocations continue to move in a similar way but to further distance. All scale bars are 200 nm.

Electron beam irradiation can displace aluminium atoms off its lattice sites, producing vacancies which may gather around dislocations with time. To identify aging effect on dislocation behaviour from irradiation-induced vacancies, a vacuum aging experiment, with and without the e-beam, was designed to check whether waiting for an extended period in vacuum in lieu of hydrogen exposure would affect dislocation motion (Supplementary Movie 1). Before the aging experiment, dislocations move between delineated positions (white dash lines at ∼27 MPa, and white solid lines at ∼250 MPa, Fig. 2a). Then the pillar was subjected to the aging process, which consisted of ∼170 min of beam-off waiting and ∼30 min of beam-on waiting at room temperature. After aging, the density of dislocation remained unchanged, although some individual dislocations underwent minor profile change (Fig. 2b,c). For example, curved segments in a dislocation may tend to relax into a straight segment, as indicated by the white arrows. This straightening of curved dislocation implies that the dislocations evolve towards a more stable configuration, possibly escaping from weaker pinning points along the dislocation line. All the dislocations moved in a similar way, and under the same loading the distance of their forward-march is larger in the first cycle after aging (Fig. 2d) compared with that in the last cycle before aging (Fig. 2a). This increased ease of movement is consistent with the trend in our measured critical activation stress in Fig. 1d, and with the straightening of curved dislocation segments. This experiment rules out the possibility that ‘aging' in vacuum and in the presence of electron irradiation could cause pinning of the dislocation lines.

**Figure 3:**
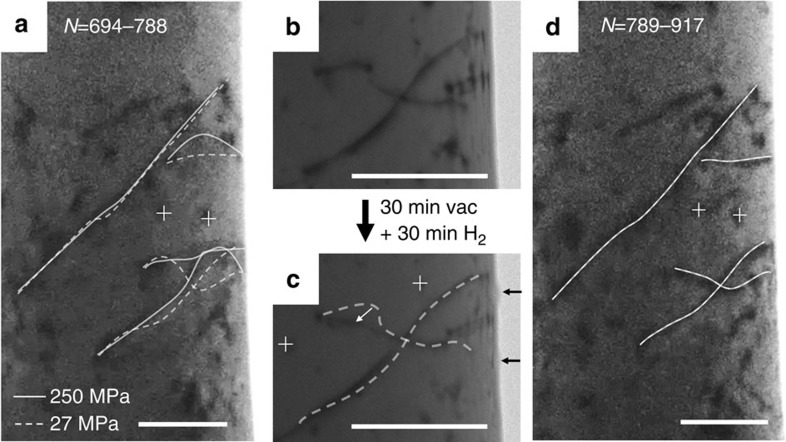
Effect of hydrogenation on dislocation movements. (**a**) Dislocation response to cyclic loads before hydrogenation. All the four observed dislocations ceased their motion immediately after hydrogenation under the same loading stress. (**b**) and (**c**) Respective positions of the four mobile dislocations before and after the hydrogenation, during which a curved segment of dislocation was relaxed due to the loss of pinning point. Besides, tiny blisters appeared on the surface (marked with black arrows). Dislocation positions in (**b**) is superimposed onto (**c**) with white dashed line for reference. (**d**) Dislocation response to the same cyclic load after hydrogenation. All dislocations ceased their motion. All scale bars are 200 nm.

As the cycle number increases, the forward-and-back movements of the dislocations became more and more reversible. After repeating such dislocation movements for additional 95 cycles (Fig. 3a), we are now in the position to watch hydrogen effects. We stopped loading, waited ∼30 min in beam-off condition in vacuum, then started the hydrogenating process, which is a ∼30 min beam-on exposure to ∼2 Pa H_2_. As seen in Fig. 3b,c, two minor changes can be observed by comparing the image before and after the hydrogenation: tiny blisters appeared on the pillar surface (as indicated by black arrows) and a short curved dislocation segment became straight (as indicated by the white arrow). The former unequivocally proves that hydrogen has been introduced into the aluminium pillar^29^. The latter, on the other hand, cannot be simply attributed to the effect of hydrogen, considering that straightening of dislocation also occurred in the previous vacuum aging experiment (Fig. 2b,c). By carefully examining this curved dislocation segment in Fig. 3b, a black pinning point can be found on the dislocation bow-out. It appears that it is the loss of this pinning point that allowed the dislocation to relax and become straight.

The key observation is that in the ensuing 85 loading cycles after hydrogenation, all five dislocations stood firm (Fig. 3d; Supplementary Movie 2), even though the loading conditions were exactly the same as that before the hydrogenation. Therefore, the critical stress to activate dislocation motion must be at least 250 MPa for all the five dislocations in the hydrogenated pillar. For dislocation #2, the critical stress is at least ∼150 MPa higher than, or 2.5 × of, the last measured value (Fig. 1d) in vacuum tests. Since vacuum aging and e-beam irradiation only tend to facilitate dislocation motion (see earlier), the observed absence of dislocation motion is due to the effect of hydrogen, which we will refer to as ‘hydrogen locking of dislocations'. The locking effect can be further corroborated by the higher critical stress for activating dislocation motion in another two different sets of experiments: monotonic compression tests (Supplementary Note 2; Supplementary Fig. 3) and bending of smooth cantilevers (Supplementary Note 3; Supplementary Fig. 4).

**Figure 4:**
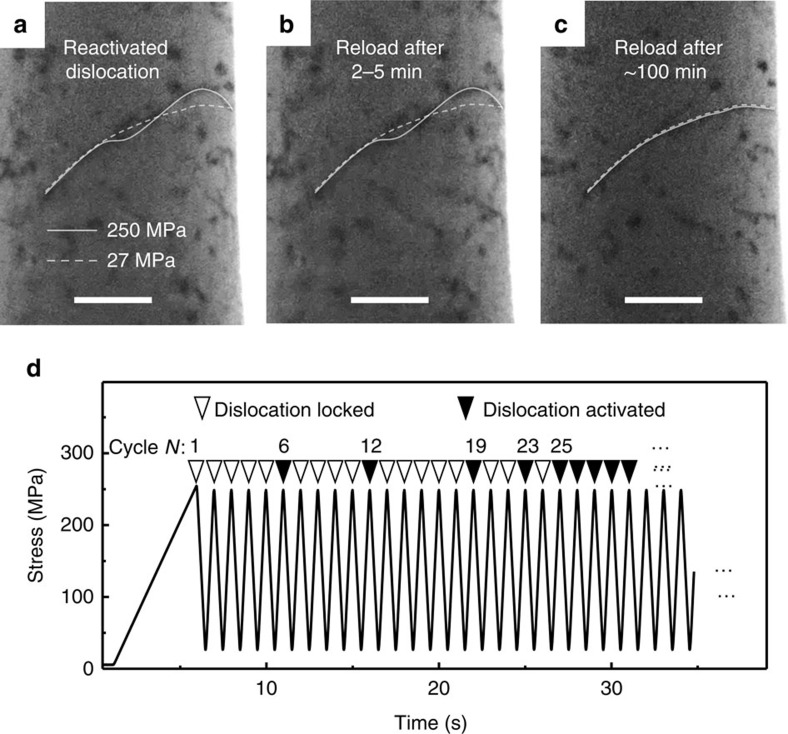
Effect of Aging time on the dislocation motion in the hydrogenated pillar. (**a**) Dislocation configuration inside the hydrogenated pillar and its dislocation movements. (**b**) After 2∼5 min aging periods, the dislocation continue moving in the same way. (**c**) After 100 min aging, the dislocation is relocked. (**d**) Dislocation movements after switching back to vacuum. The intermittent activation of dislocation motion shows an increasing chance of reoccurrence, as the hydrogen content diminishes with time. All scale bars are 200 nm.

Later in the experiments these reactivated dislocations changed their profiles dramatically due to a drift of the loading punch, which induced buckling and exerted an additional stress on dislocations (Supplementary Note 4; Supplementary Fig. 5). The dislocation configuration inside the pillar was re-stabilized by continuing to cycle with a peak stress of ∼250 MPa for 81 cycles, and at this time only one long mobile dislocation was left. As shown in Fig. 4a, this dislocation does the same reversible movements for 186 cycles. These cycles are from 9 tests, and between each two successive tests there is a resting time of 2–5 min for tip realignment and sample characterization. It is surprising to observe that during these periods, hydrogen has never re-gathered to re-lock the dislocation to stop its subsequent motion, as shown in Fig. 4b.

The hydrogen gas was then cut off and the pillar was aged in vacuum for about 100 min, to allow the redistribution of the trapped hydrogen. After this step, the same cycling load was applied to the pillar (Supplementary Movie 3). It can be seen that the dislocation has been re-locked, as seen in the first few cycles in Fig. 4c. But in the following cycles, movement was initiated in a stick-slip manner, as marked by black triangles in Fig. 4d. The first movement happened in the sixth cycle of this test, but the next movement happened six cycles later. As the cycle number increases, the intermittent dislocation movement becomes more and more frequent as the dislocation shed obstacles and eventually it turns into successive reciprocating motion in each cycle, for all cycles after *N*=25.

To recapitulate, our cyclic compression tests above have provided clear evidence that dislocation locking will take place in the presence of H. However, the long diffusion time of a few tens of minutes indicates that the observed locking effect is not from H interstitials acting alone. This conjecture arises because in some *in situ* TEM experiments, hydrogen introduction is reported to impact dislocation behaviour in less than one second^12^,^30^. The enthalpy of migration of hydrogen interstitial in aluminium is estimated to be only about 0.19 eV (refs 31, 32); this translates into reaching equilibrium within one second in a sub-micron sample. Such hydrogen diffusion times are at least three orders of magnitude less than that in our experimental observation. Therefore, the strong locking of dislocations should involve other forms of point defects. As mentioned before, superabundant vacancies are likely preserved inside metals, no matter whether the hydrogen charging methods are chemical, electrochemical or plasma based^16^,^33^. In our experiments, the incident electron beam interacts with and ionize the hydrogen gas. The produced free hydrogen atoms and ions make the local environment quite similar to the hydrogen plasma reported by Buckley *et al*.^16^, such that after hydrogen exposure there should be superabundant vacancies inside the aluminium pillar. The diffusion should then involve the migration of hydrogen-vacancy complexes, which are energetically more stable and also move much more sluggishly than hydrogen interstitials^34^,^35^,^36^. Such gas-stabilized vacancies and cavities are indeed well known in radiation damage^37^, which also cause plastic flow localization and embrittlement. Therefore, we propose that it is the segregation of hydrogenated vacancy to the dislocation core that has resulted in the observed dislocation locking.

### Atomistic simulations

**Figure 5:**
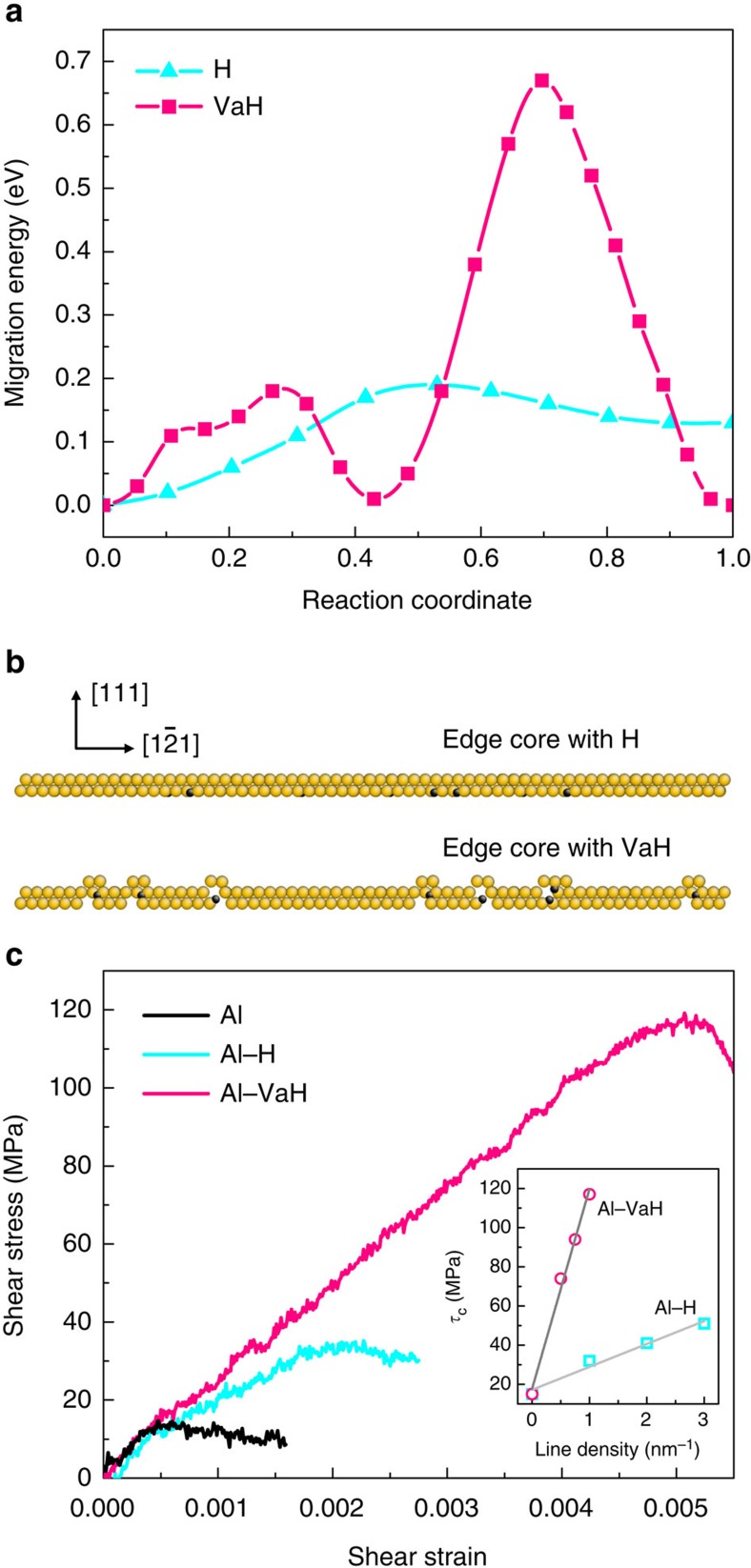
Atomistic simulation of the pinning effect of hydrogen-vacancy complexes on dislocation motion. (**a**) Migration paths of hydrogen (H) from one tetrahedral (T) interstitial site to the neighbouring octahedral (O) site, and hydrogen-vacancy (VaH) complexes in aluminium. (**b**) Side view of dislocation core decorated by hydrogen and hydrogen-vacancy complex, respectively. Atoms with golden and black colours refer to aluminum and hydrogen, respectively. (**c**) The shear stress-strain curves of different systems. The density of hydrogen and hydrogen-vacancy complexes along dislocation line is one per nanometre. The inset shows the dependence of critical shear stress (*τ*_c_) on the concentration of hydrogen and hydrogen-vacancy complexes. The linear fitting is made for both systems.

Earlier density functional theory calculations have shown that the binding energy of one H interstitial and one vacancy (Va) is around 0.4 eV (ref. 38), which indicates that the hydrogen-vacancy (VaH) complex is relatively stable. Based on a Al-H angular-dependent potential (ADP)^39^ (see Methods and Supplementary Note 5 for the evaluation of this potential), we calculated the migration paths of hydrogen and hydrogen-vacancy complex by using the climbing image nudged elastic band method^40^, as shown in Fig. 5a. In comparison with the barrier of 0.19 eV for H interstitial diffusion, a VaH complex needs to overcome a much higher barrier of 0.67 eV. We can then obtain the diffusion coefficient of the VaH complex at room temperature as *D*=*D*_0_exp(−*E*^m^/*k*_B_*T*)≈2.8 × 10^−14^ cm^2^ s^−1^, where *D*_0_=5.9 × 10^−7^ m^2^ s^−1^ (ref. 41), *E*^m^ is the diffusion barrier, *k*_B_ is Boltzmann constant and *T* is absolute temperature. In agreement with this, the diffusivity of hydrogen–vacancy complexes was reported to be of the magnitude of 10^−15^ cm^2^ s^−1^ in pure aluminium^16^,^31^. Finally, we can estimate the time scale for the hydrogen–vacancy complex to diffuse from the surface to the centre of pillar, *t*=*r*^2^/4*D*, where *r* is the pillar radius (300 nm). The diffusion time required is ∼120 min at room temperature; this is consistent with the locking time scale seen in our experiments.

In comparison to that of hydrogen interstitials (Supplementary Note 6; Supplementary Figs 6–7), we further simulate the effect of hydrogen-vacancy complexes on the critical stress of dislocation motion (Supplementary Note 7; Supplementary Figs 8–9). Figure 5b shows the configurations of edge dislocation core decorated with hydrogen interstitials and hydrogen–vacancy complexes, respectively. The hydrogen interstitials do not change the core structure of dislocation very much, while hydrogen–vacancy complexes can form dislocation jogs. Figure 5c shows the shear stress-strain curves for pure Al, Al–H, and Al–VaH systems, under the same level of segregation with one H (or VaH) per nanometre in the dislocation line. Compared with pure Al, a higher shear stress (∼32 MPa) is required for driving dislocation motion, indicating that hydrogen interstitials take certain pinning effect on dislocation motion. In contrast, for Al–VaH, the critical shear stress (∼117 MPa) is even much higher than that for Al–H. This suggests a much stronger locking effect from VaH. Our recent studies also showed that the hydrogen–vacancy complex roots very stably in the lattice when meeting dislocations in BCC α-Fe (ref. 18), instead of being picked up and swept away by the moving dislocation. Here the simulations in Al further confirm that such complex can hinder dislocation motion strongly in FCC metals. Moreover, the critical shear stress (*τ*_c_) for dislocation motion is found to increase linearly with the concentration of hydrogen and hydrogen-vacancy complexes along the dislocation core (inset in Fig. 5c), and in the case of hydrogen–vacancy complex shows a much steeper slope.

## Discussion

Evidence of intense dislocation activity can be found in many metals that show macroscopic HE, at least locally at ∼10^2^ nm length scale beneath the fracture surface^10^,^20^. Stress-driven dislocation activities, such as intersecting of two dislocations, are known to produce excess vacancies atomistically (now further stabilized by H)^18^,^20^, forming a feedback loop of ever-more intense localization. In non-hydride-forming metals, hydrogen-enhanced localized plasticity (HELP)^11^,^42^ as a descriptive mechanism finds widespread support. According to the HELP theory of HE (ref. 10), plastic localization happens first, and main material damage happens later^20^, in a fashion like ductile failures in metals without hydrogen, but within a reduced space-time volume, giving rise to reduced fracture toughness overall. Plastic localization requires microscopic mechanisms for softening and/or flow planarization. While not addressing the entire question, we note certain parallels between what we see in our ETEM measurements, namely VaH locking and time-dependent stress-driven unlocking of dislocation, and strain aging induced plastic flow localization. So while a concentrated VaH atmosphere hardens the material initially, it also sets it up for a potentially dramatic softening later, if the dislocation cores shake free of the segregated VaH by moving long enough distances without stopping, which we have directly seen in our ETEM experiment. This dynamic softening is expected to be the most active near a macro-crack tip, where the tensile triaxial stress is the greatest to attract the largest hydrogen background concentration. So strain aging induced plastic localization instability may occur in hydrogen-charged metal^43^,^44^, due to the hysteretic dislocation motion. Once an initial strain-softening and plastic localization length scale is established, the stored elastic energies of the surrounding materials will be dumped into this zone, greatly accelerating material damage accumulation in the forms of more hydrogenated vacancies and cavities^18^,^20^, cracking of interfaces, etc. which may activate secondary mechanisms of softening and further localization, until the damaged material (defined by reduced total metal–metal coordination number) is localized to sharper and sharper zones, ultimately turning into two topologically separate surfaces. Our finding of time-dependent dislocation locking/unlocking due to hydrogen-vacancy complexes, observed at individual dislocation level in ETEM, sheds light on the initiation and feedback mechanisms of HELP and HE in non-hydride-forming metals. It likely also has bearing on subsequent damage accumulation, since vacancy is the smallest unit of structural damage as measured by reduction of total metal–metal coordination (for example gas-stabilized vacancies and cavities are basic forms of radiation damage^37^), a supersaturation of which can be easily generated by the plethora of dislocation intersections in a region with heavy plastic deformation^18^,^20^.

## Methods

### Sample preparation

Single crystal aluminium (99.9995%) disks were cut into 1.5 × 2 mm^2^ rectangular plates, which were mechanically polished to 100 μm in thickness and electrochemically thinned to a few microns at one edge. Submicron-sized cylindrical pillars were prepared by Focused ion beam (FIB, FEI Helios NanoLab 600, operating at 30 keV) milling on the thinned edge. The used milling current in the last step was as low as ∼20 pA to minimize geometrical taper and irradiation damage induced by ion beam. The thickness of surface affected layers were about 7 nm according to the TEM observation. All pillars had a taper of ∼2.5° and an aspect ratio (top diameter/length) of ∼1/3.

### *In situ* mechanical tests

The *in situ* TEM nanocompression tests were performed using a Hysitron PI95 H1H PicoIndenter. Pillars were loaded using a diamond punch, which was connected to a MEMS transducer with force resolution of ∼300 nN and displacement resolution of ∼2 nm. Both the loading direction and electron-beam direction were 〈110〉 direction for all the tested pillars (Supplementary Fig. 1). A pillar of 620 nm in top diameter was used for cyclic compression test. A carbon cap layer of 100 nm in thickness was deposited atop the aluminium by ion beam assisted deposition under beam current of 18 pA, as shown in Fig. 1a. This cap layer has low adhesion force to the diamond punch and can help to maintain a stable contact between the punch and pillar during multiple tests. At the same time, this cap layer can act as a buffer layer, which can help avoid unnecessary dislocation emission from the top surface by eliminating stress singularities, so that dislocation configuration inside the pillar can remain undistracted after many tests.

We used load control since the feedback control can automatically compensate for the displacement drift in the loading direction and assure that all cycles reach almost the same peak stress. The whole experiment consists of 21 loading sessions, and each loading session has 6–99 cycles, of which the leading cycle is 5 s (4.5 s loading+0.5 s unloading) and all the trailing cycles are 1 s per cycle (0.5 s loading+0.5 s unloading). In total, 1414 cycles were applied to the sample. During cyclic compression, the loading stress oscillates between valley stress (25–30 MPa) and peak stress (∼330 or ∼250 MPa), as shown in Fig. 2b. The first 339 cycles use peak stress of ∼330 MPa to mechanically decrease the dislocation density, and all the other cycles have peak stress of ∼250 MPa to manipulate the movements of the few mobile dislocations left.

Real-time observation was conducted in an environmental TEM (Hitachi H9500, operating at 300 kV and 1 μA emission current). The compression tests were carried out subsequently in vacuum and ∼2 Pa pure hydrogen gas. Before the tests in hydrogen environment, samples were hydrogenated under electron-beam intensity of ∼0.45 nA μm^−2^ in ∼2 Pa H_2_ for about 30 min, which is long enough for hydrogen diffusion while not too long to cause obvious blistering on the pillar surface^29^. The dislocations inside the pillar roughly keep the same configuration during the charging process. And the following mechanical tests were performed in the same hydrogen atmosphere but with much lower beam intensity (∼0.05 nA μm^−2^).

### Atomistic simulations

The interatomic interaction in Al–H system is described by an angular dependent empirical potential^39^. This potential reproduces many properties quite well, especially the energetics related to the point defects such as hydrogen dissolution energy, hydrogen migration energy and so on, but it underestimates the binding energy of hydrogen–vacancy complex (Supplementary Note 5; Supplementary Tables 1–3). To address the kinetic properties of the H and VaH complex, the migration barriers and paths were calculated by using the climbing image nudged elastic band^40^ method. We further studied the locking effect that hydrogen or hydrogen-vacancy complexes exert on dislocation motion. Although the binding energy of VaH is underestimated by ADP potential, we did not use the ADP to calculate H, Va and VaH partition in H+Va=VaH. We simply placed VaH complexes inside the dislocation core, and study their dragging effect on the dislocation. For this particular calculation, the binding energy of H with Va to form VaH is mostly irrelevant. The binding energy of the dislocation core with VaH is more important.

For the simulations, we first created an *a*/2〈110〉-type edge dislocations in Al. The simulation box was oriented along *x*-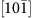, *y*-[111], *z*-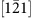, with dimensions of 23 × 28 × 16 nm^3^. Periodic boundary conditions were applied in the *x* and *z* directions. A certain amount of hydrogen atoms or hydrogen-vacancy complexes were then introduced in the region around the dislocation core regime according to the Boltzmann occupation probability. The system was further relaxed at 300 K for 100 ps using a Nosé-Hoover thermostat^45^. Finally shear strain was applied in the *xz*-plane to drive dislocation glide along the *x* direction. The strain rate was on the order of 10^8^ s^−1^. The whole calculations were carried out using the LAMMPS (ref. 46) code and the atomic configurations were displayed via AtomEye^47^. The details of model construction and simulation procedure are shown in Supplementary Notes 6 and 7.

### Data availability

The data that support the findings of this study are available from the corresponding authors on request.

## Additional information

**How to cite this article**: Xie, D. *et al*. Hydrogenated vacancies lock dislocations in aluminium. *Nat. Commun.* **7**, 13341 doi: 10.1038/ncomms13341 (2016).

**Publisher's note:** Springer Nature remains neutral with regard to jurisdictional claims in published maps and institutional affiliations.

## Supplementary Material

Supplementary Information Supplementary Figures 1 - 9, Supplementary Tables 1 - 3, Supplementary Notes 1 - 7 and Supplementary References

Supplementary Movie 1 Effect of vacuum aging process on dislocation motion for the hydrogen-free pillar.

Supplementary Movie 2 Effect of hydrogenation on dislocation motion

Supplementary Movie 3 Effect of vacuum aging on dislocation motion of the hydrogenated pillar.

Peer Review
