# Supplementary material for: Hydrogenated vacancies lock dislocations in aluminium
Source: Nat Commun. 2016 Nov 3;7:13341. doi: 10.1038/ncomms13341 (PMC5097162; doi:10.1038/ncomms13341)
Supplement: Supplementary Information — Supplementary Figures 1 - 9, Supplementary Tables 1 - 3, Supplementary Notes 1 - 7 and Supplementary References [file ncomms13341-s1.pdf]

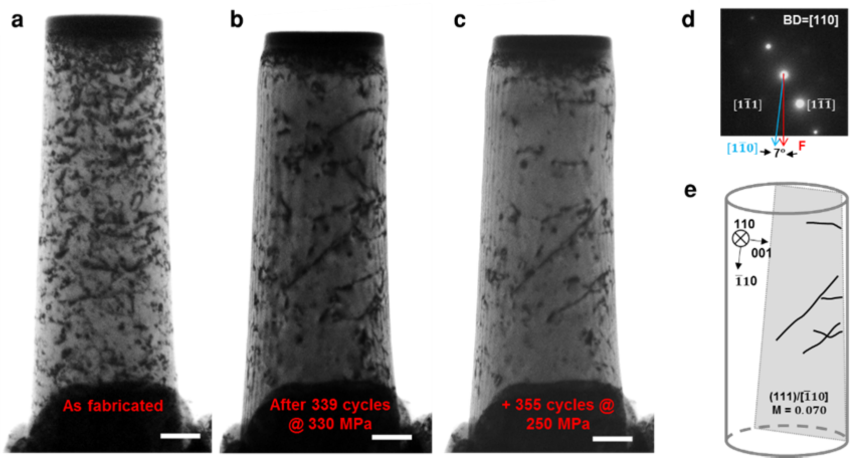

Supplementary Figure 1. Process to achieve individual-dislocation configuration through small-strain-amplitude cyclic compression. (**a)** As-fabricated pillar contains high density of dislocations. (**b)** After 339 cycles of cyclic compression with peak stress of 330 MPa, the dislocation density dramatically decreased. (**c)** After another 355 cycles of cyclic compression with peak stress of 250 MPa, only a few long dislocations remained inside the pillar. (**d**) The diffraction pattern of the single crystal aluminum pillar, for which the loading axis is inclined to the $[1\bar{1}0]$ direction by 7°. (**e**) Schematic of the mobile dislocations in (c) and one of the possible slip system, for which the Schmidt's factor M is calculated to be 0.070. All scale bars represent 200 nm.

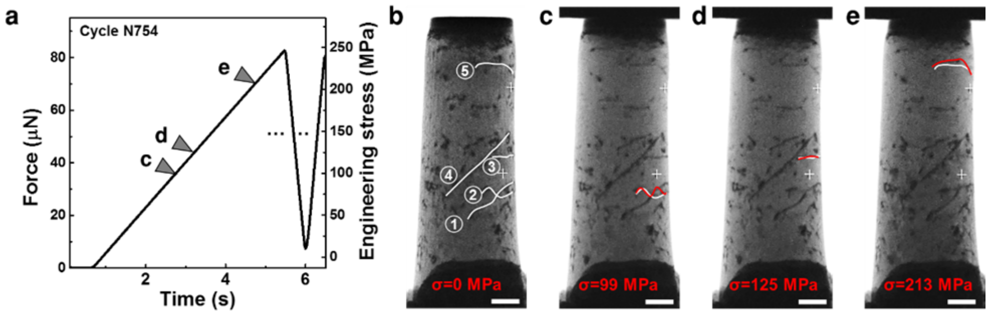

Supplementary Figure 2. The threshold loads for each dislocation determined in the first loading cycle in vacuum. (**a)** Load vs. time curve of the slow leading cycle. The triangular markers correspond to the threshold loads of each dislocation to be activated. (**b)** The original configuration of the dislocations in the pillar before loading. (**c)**, (**d)**, and (**e)** show the abrupt jump of dislocations #2, #3 and #5 at critical loading stresses of 99, 125, and 213 MPa respectively. The red lines and white lines are the dislocation profiles before and after the jump. For more details see supplementary Movie 1. All scale bars represent 200 nm.

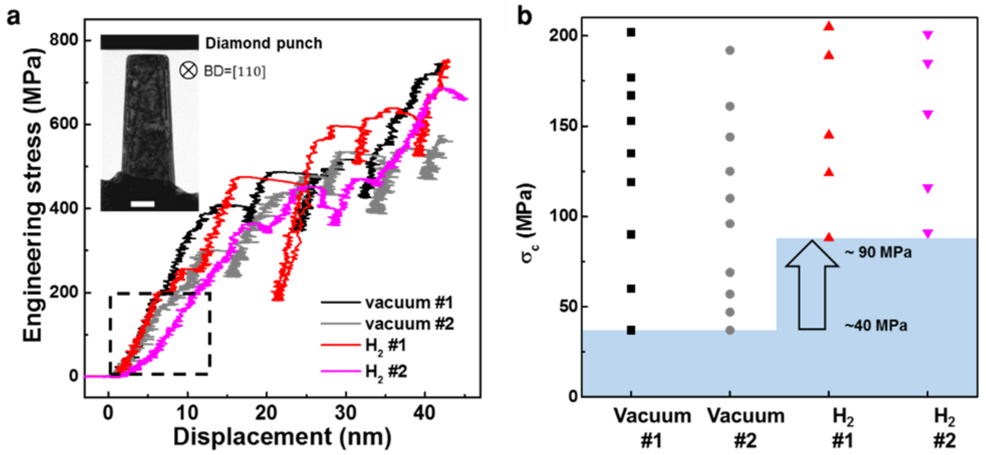

Supplementary Figure 3. Monotonic compression of aluminum pillars in vacuum and hydrogen gas. (**a**) Engineering stress-displacement curves showing similar yield stress and flow stress for tests in vacuum and in hydrogen. The inset illustrates the experimental setup. (**b**) The critical stress (σ_c) for dislocation depinning events in various samples, showing that a higher stress is required to set off dislocation motion in hydrogenated samples. Scale bar in inset in (a) is 200 nm.

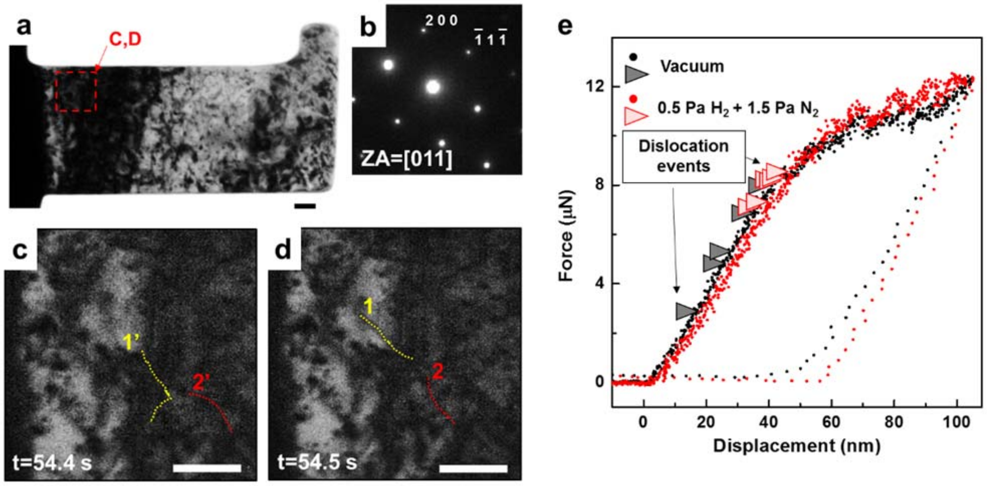

Supplementary Figure 4. Hydrogen effect on dislocation motion during bending tests. (**a**) Bright field TEM image of a typical bending cantilever containing dislocations and other defects. (**b**) The corresponding diffraction pattern showing the single crystal nature of the cantilever. (**c**) and (**d**) are the magnified area in (**a**) showing two typical dislocation profile at the given moments before and after sudden movements. (**e**) Force vs. displacement curves of two typical samples with similar dimensions but tested in vacuum and hydrogen environment, respectively. The first 5 dislocation events of each test are labeled on the curve with triangle marks. All scale bars represent 100 nm.

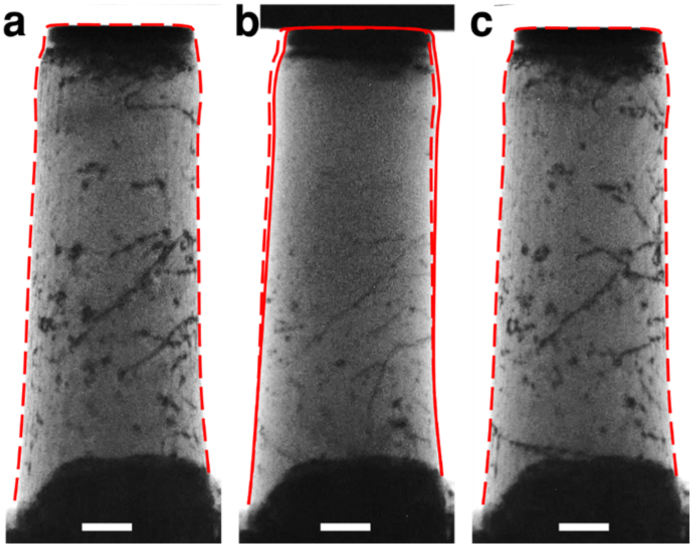

Supplementary Figure 5. Destruction of dislocation configuration due to bucking during the test in 2 Pa H_2. (**a**) The dislocation configuration inside the pillar remained unchanged after 85 cycles. (**b**) Due to the tip drift, the pillar was buckled. The dislocation configuration underwent a sudden change under the coupling of loading stress and buckling stress. Note pillar contour change before drift (red dashed line) and after drift (red solid line), as well as the change of black contrast inside the pillar due to buckling. (**c**) When the tip is detached from the pillar, the pillar contour recovered but the dislocation configuration can’t be resumed. All scale bars represent 200 nm.

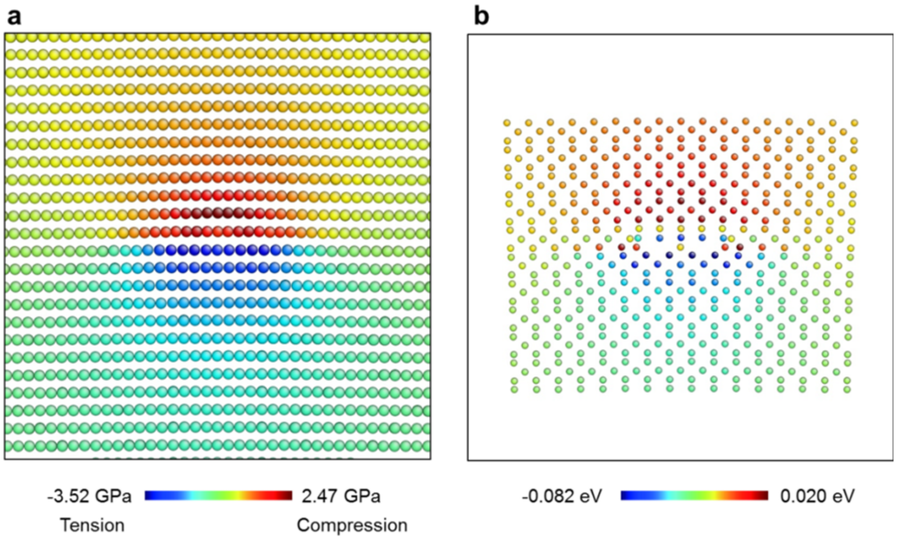

Supplementary Figure 6. The distribution maps of (**a**) hydrostatic stress of edge dislocation in aluminum and (**b**) total energy difference of hydrogen ($\Delta E_{H,i}$) in interstitial sites around the dislocation core. Generally, interstitial hydrogen in the region with minus hydrostatic stress (tension) has a smaller value of $\Delta E_{H,i}$ and that in the region with positive hydrostatic stress (compression) has a larger value of $\Delta E_{H,i}$. The tensile region around dislocation core corresponds to the most energy-favored sites for hydrogen atoms.

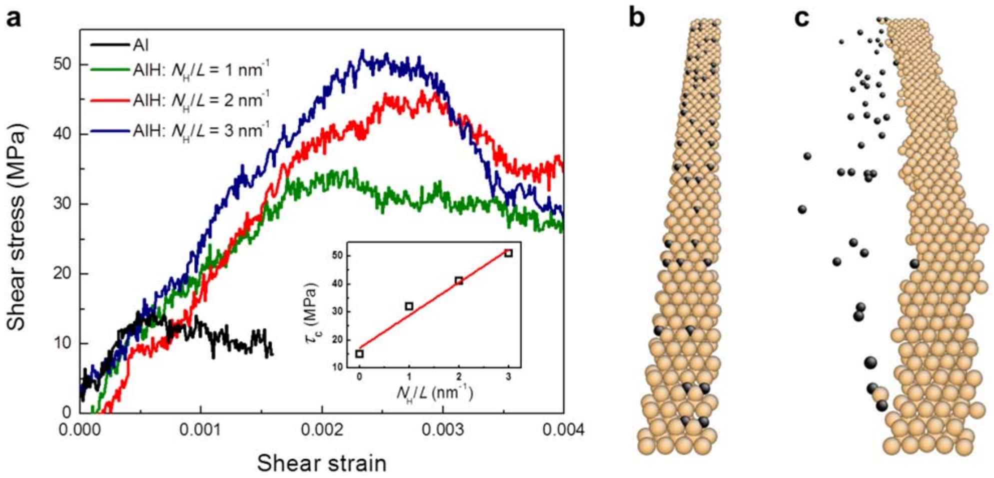

Supplementary Figure 7. Atomistic simulations based on the model with hydrogen atoms in a Boltzmann distribution. (**a**) Stress-strain curves at different hydrogen concentration levels. The critical shear stress (τ_c) for dislocation motion shows a linear increase with the increase of hydrogen concentration (N_H/L), as shown in the inset. (**b**) Initial configuration of dislocation decorated with hydrogen atoms. (**c**) The configuration at the depinning state. Atoms with golden and black colors refer to aluminum and hydrogen.

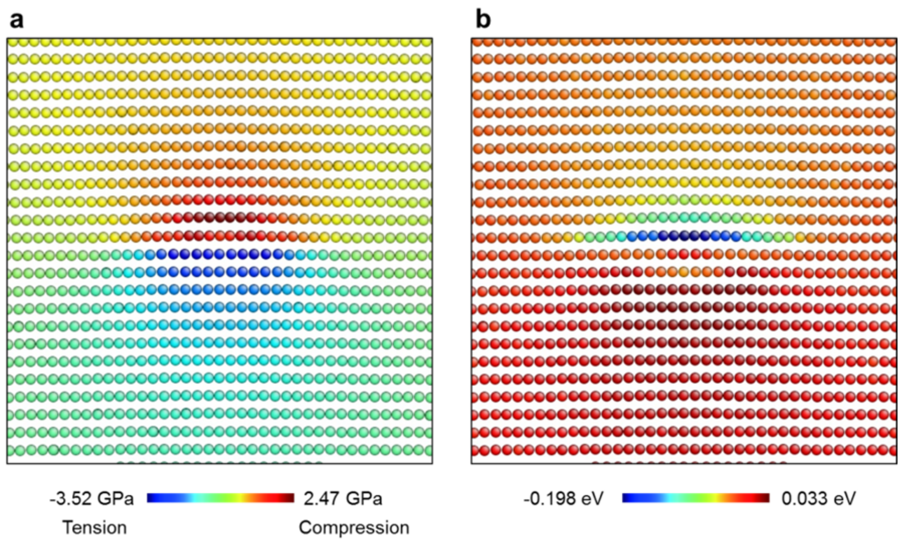

Supplementary Figure 8. The distribution maps of (**a**) hydrostatic stress of edge dislocation in aluminum and (**b**) total energy difference of VaH complex ($\Delta E_{\mathrm{VaH},i}$) in site i ($E_{\mathrm{VaH},i}^{\mathrm{Edge}}$) with reference to that in bulk site ($E_{\mathrm{VaH},i}^{\mathrm{Bulk}}$).

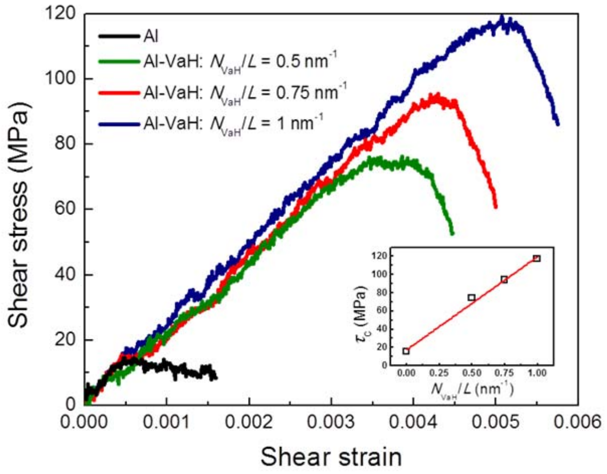

Supplementary Figure 9. MD simulations of dislocation pinning effect by hydrogen-vacancy complex. The different colored lines show the stress-strain curves at different concentrations of hydrogen-vacancy complex. The critical shear stress (τ_c) for dislocation motion shows a linear increase with the increase of hydrogen-vacancy complex concentration (N_{VaH}/L), as shown in the inset.

Supplementary Table 1. Energetics of isolated H atom in Al. ΔE_s refers to the solution energy of H in Al. T and O refer to the tetrahedral (T) interstitial and octahedral (O) interstitial sites. E_m refers to the migration barrier of one H atom from a T-site to the O-site. The units are in eV.

	ADP	ab initio
ΔE_s (T)	0.69	0.69 (ref. 1)
ΔE_s (O)	0.82	0.82 (ref. 1)
E_m	0.19	0.18 (ref. 1)

Supplementary Table 2. Energetics of Va in Al. E_v^f refers to the vacancy formation energy and E_m refers to the vacancy migration barrier. The units are in eV.

	ADP	Experiments
E_v^f	0.68	0.69 (ref. 2)
E_m	0.64	0.65 (ref. 3)

Supplementary Table 3. Energetics of VaH. E_f (T-Va) refers to the formation energy of vacancy with H in T-site. E_f (O-Va) refers to the formation energy of vacancy with H in O-site. E_b (T-Va) refers to the binding energy of vacancy with H in T-site. The units are in eV.

	ADP	ab initio
E_f (T-Va)	1.32	1.0 (ref. 4)
E_f (O-Va)	1.36	1.14 (ref. 4)
E_b (T-Va)	0.048	0.39 (ref. 5)

Supplementary Note 1: <u>Mechanical annealing by cyclic compression</u>

The loading function consists of a slow leading cycle and tens of fast trailing cycles in each group of test. In the loading half-cycle, each dislocation marches forward, like a bending bow, with both ends pinned on surface; upon unloading, each swings back to the original location (see supplementary Movie 1).

The initial compression tests were conducted with peak stress of ~330 MPa. By the end of 339th cycle (N339), only a few isolated mobile dislocations were left, with their both ends pinned by the surface of the sample (Supplementary Fig. 1b). In order to stabilize these dislocations further, we purposely lowered the peak stress down to ~250 MPa. A steady dislocation configuration was reached at N694 (Supplementary Fig. 1c), i.e. for each following cycle, the bow out extent of each dislocation remained the same at each peak stress.

In Supplementary Fig. 2, three dislocations show abrupt position change during the loading, whereas the two other dislocations only show smooth position change. Critical loading stress for dislocation #1, #3, and #5 are 99 MPa, 125 MPa, and 213 MPa respectively.

Supplementary Note 2: <u>Monotonic compression</u>

Four typical engineering stress-strain curves from monotonic compression tests are shown in Supplementary Fig. 3. Before the stress reaches 200 MPa, the curve looks linear without obvious yielding or strain burst. But when correlated with the movie frames, discrete movements of preexisting dislocations can be observed. These preexisting dislocations in the as-fabricated pillars mainly result from FIB damage in the milling process, and are pinned by obstacles such as surface junctions, point defects and other dislocations. As the applied load increases, dislocations start breaking away from pinning points, exhibiting abrupt movements. After depinning,

they either vanish at surfaces or get stopped at a stronger pinning point. We have tracked all the observed depinning events and their corresponding critical stresses, and found that the lowest depinning stress increases from ~40 MPa to ~90 MPa when changing the testing environment from vacuum to the 2 Pa hydrogen gas, as demonstrated in Supplementary Fig. 3b.

From the engineering stress-displacement curves, monotonic compressions in hydrogen environment show similar stiffness and strain hardening behavior when compared to compressions in vacuum. In these tests, the profile of stress-strain curve is mainly governed by sample-size dependent dislocation nucleation and multiplication. In Supplementary Fig. 3a, H_2 #2 specimen show lower stiffness than the other three pillars due to the slight misalignment and initial point contact at the beginning of the compression test.

Supplementary Note 3: <u>Hydrogen effect on dislocation motion in bending tests</u>

8 smooth cantilevers with width ranging from 222 nm to 292 nm and thickness from 600 nm to 642 nm were bent inside the ETEM. All the cantilevers (e.g. Supplementary Fig. 4a) contained preexisted dislocations resulting mainly from FIB milling. The dislocation density was estimated to be between 10^{13} m^{-2} and 10^{14} m^{-2}. Monotonic bending loads were applied to the cantilevers at loading rate of 5 nm/s under displacement control mode. Total 8 samples were tested, 4 in vacuum and 4 in hydrogen environment. Similar to that observed in monotonic compression tests (Supplementary Fig. 3), the threshold loads for dislocation activation in the hydrogen atmosphere are always higher than those tested in vacuum. One typical comparison is shown in Supplementary Figure 4e. It again demonstrates that hydrogen can improve the threshold loads significantly for those preexisted dislocations.

Supplementary Note 4: Reconfiguration of dislocations due to buckling/bending induced by probe drift

Since a typical cyclic loading test lasts 40 to 100 seconds, the diamond punch may undergo obvious drift in all three space coordinate directions. Under the load control system, drift in the indentation direction can be automatically compensated by the feedback control system so as to ensure a correct loading force applied to the pillar. However, drifts perpendicular to the indentation axis can buckle the samples obviously due to the friction confinement at the contact interface between the diamond tip and the samples. This will apply addition stress to the dislocations. One typical example is shown in Supplementary Fig. 5. The sample was bended/buckled obviously (Supplementary Fig. 5b) during the cyclic loading, as suggested by the profile deviation (red dashed lines) and the significant contrast change. New dislocations coming out from the bottom disrupted the dislocation configuration inside the pillar. Interestingly, the sample can go back to its original position after the diamond probe was retracted despite that the dislocation configurations had been dramatically changed.

Supplementary Note 5: Evaluation of Al-H angular dependent potential

To provide more details about the empirical angular-dependent potential (ADP)[6] we used in MD simulations, here we show various properties predicted by this potential in comparison to the experiments and ab initio calculations, especially some energetics related to the point defects (H, Va and VaH), as shown in the following three Tables 1, 2 and 3. Among them, most has a relatively good match with the referenced data, but unfortunately, the binding energy of VaH is very small. More information for this potential is shown in refs 6, 7.

Supplementary Note 6: Molecular dynamics simulation of the influence of hydrogen atmosphere on dislocation motion

We first created the $a/2\langle 110\rangle$-type edge dislocations in Al. The simulation box was oriented along x-$[10\bar{1}]$, y-$[111]$, z-$[1\bar{2}1]$, with dimensions of 23nm × 28nm × 16nm. Periodic boundary conditions were applied in the x and z directions. The hydrogen atoms were then introduced in the interstitial tetrahedral sites around the dislocation according to the Boltzmann occupation probability

$$p_{\mathrm{H},i} = e^{\Delta E_{\mathrm{H},i}/k_{\mathrm{B}}T} / \sum_i e^{\Delta E_{\mathrm{H},i}/k_{\mathrm{B}}T} \tag{S1}$$

where $\Delta E_{\mathrm{H},i} (= E_{\mathrm{H},i} - E_{\mathrm{H},i}^{\mathrm{Bulk}})$ is the total energy difference of hydrogen in site i ($E_{\mathrm{H},i}$) with reference to that in bulk tetrahedral interstitial site ($E_{\mathrm{H},i}^{\mathrm{Bulk}}$), k_{B} is Boltzmann constant and T is absolute temperature. Supplementary Fig. 6a shows the distribution of hydrostatic stress of the edge dislocation and Supplementary Fig. 6b shows the distribution of $\Delta E_{\mathrm{H},i}$ in tetrahedral site around the dislocation core. We can see that the two maps match relatively well. The tensile region corresponds to a much lower energy state, where most of the hydrogen atoms distribute. The most energy-favored sites for hydrogen are right in the dislocation core region. The hydrogen concentration around dislocation line is characterized with N_{H}/L, where N_{H} is the number of hydrogen atoms and L is the length of the dislocation line[8]. After adding hydrogen atoms, the system was further relaxed at 300 K for 100 ps using a Nosé-Hoover thermostat[9, 10]. Finally shear strain was applied in the xz-plane to drive dislocation glide along the x direction. The strain rate was on the order of 10^8 s^{-1}. The whole calculations were carried out using the LAMMPS code[11] and the atomic configurations were displayed via AtomEye[12].

Supplementary Fig. 7a shows the shear stress-strain curves for Al-H system in comparison to H-free system at 300 K. An obvious locking effect can be observed: the

value of critical shear stress for edge dislocation motion increases in hydrogen environment. The dependence of locking strength on hydrogen concentration is studied by conducting a serial of simulations with different N_H/L. The inset in Supplementary Fig. 7a shows the variation of critical shear stress (τ_c) with different amount of N_H/L. We find that the value of τ_c increases almost linearly with hydrogen concentration as

$$\tau_c = \tau_0 + \alpha N_H / L \tag{S2}$$

where τ_0 (= 15 MPa) is the critical shear stress for dislocation motion without hydrogen and the coefficient α is 11.7 MPa·nm in the present Al-H system. It is interesting to observe that even hydrogen with a density of N_H/L = 1 nm^{-1} could double the τ_c. Supplementary Fig. 7b shows the initial configuration of dislocation decorated with hydrogen atoms at the level of N_H/L = 3 nm^{-1}. When the shear stress reaches the critical value, the dislocation suddenly escapes from the hydrogen atmosphere, as illustrated in Supplementary Fig. 7c.

Our MD simulations reveal in a straightforward manner that hydrogen atoms could enhance the critical stress for dislocation motion. The interaction between dislocation and diffusing solute hydrogen atoms can lead to locking effect, similar to that in strain aging[13, 14].

Supplementary Note 7: <u>Molecular dynamics simulation of the influence of hydrogen-vacancy complexes on dislocation motion</u>

Our simulations above show that the hydrogen atoms themselves can take the pinning effect on dislocation motion. However, as we mentioned in the manuscript, the aging experiments show that the relocking needs a long time beyond hydrogen diffusion time. It is reasonable to speculate that there are some other obstacles to lock dislocations. By calculating the migration energy and estimating the diffusion length, we found that the most probable candidate is hydrogen-vacancy complex. In the

following, another set of simulations was performed to reveal the role of hydrogen-vacancy complex on dislocation motion. Similar to that in Al-H system, we introduced the VaH complexes according to the Boltzmann occupation probability

$$p_{\mathrm{VaH},i} = e^{\Delta E_{\mathrm{VaH},i}/k_{\mathrm{B}}T} / \sum_{i} e^{\Delta E_{\mathrm{VaH},i}/k_{\mathrm{B}}T} \quad \text{(S3)}$$

where $\Delta E_{\mathrm{VaH},i}$ is the total energy difference of VaH complex in site i ($E_{\mathrm{VaH},i}$) with reference to that in bulk site ($E_{\mathrm{VaH},i}^{\mathrm{Bulk}}$). Supplementary Fig. 8a shows the distribution of hydrostatic stress of the edge dislocation and Supplementary Fig. 8b shows the distribution of $\Delta E_{\mathrm{VaH},i}$ around the dislocation core. Supplementary Fig. 9 shows the shear stress-strain curves at different concentrations of hydrogen-vacancy complexes. It can be seen that the more hydrogen-vacancy complexes are implanted, the stronger pinning effect will be. The critical shear stress (τ_c) for driving dislocation motion also shows a linear response with the line density of hydrogen-vacancy complex (N_{VaH}/L), but with a much steeper slop (101.9 MPa·nm) than that pinned by hydrogen. Our current simulations show that the hydrogen-vacancy complex take an even stronger pinning role than the hydrogen atmosphere.

Supplementary References

1. Wolverton C, Ozoliņš V, Asta M. Hydrogen in aluminum: First-principles calculations of structure and thermodynamics. *Phys. Rev. B* **69**, 144109 (2004).
2. Schaefer HE, Gugelmeier R, Schmolz M, Seeger A. Positron lifetime spectroscopy and trapping at vacancies in aluminium. *Mater. Sci. Forum* **15-18**, 111-116 (1987).
3. Balluffi R. Vacancy defect mobilities and binding energies obtained from annealing studies. *J. Nucl. Mater.* **69**, 240-263 (1978).
4. Wolverton C, Ozoli?? V, Asta M. Hydrogen in aluminum: First-principles calculations of structure and thermodynamics. *Phys. Rev. B* **69**, 1124-1133 (2004).
5. Lu G, Kaxiras E. Hydrogen embrittlement of aluminum: The crucial role of vacancies. *Phys. Rev. Lett.* **94**, 155501 (2005).

6. Apostol F, Mishin Y. Angular-dependent interatomic potential for the aluminum-hydrogen system. *Phys. Rev. B* **82**, 144115 (2010).

7. Mishin Y, Farkas D, Mehl MJ, Papaconstantopoulos DA. Interatomic potentials for monoatomic metals from experimental data and ab initio calculations. *Phys. Rev. B* **59**, 3393-3407 (1999).

8. Song J, Curtin WA. Mechanisms of hydrogen-enhanced localized plasticity: An atomistic study using α-Fe as a model system. *Acta Mater.* **68**, 61-69 (2014).

9. Hoover WG. Canonical dynamics: equilibrium phase-space distributions. *Phys. Rev. A* **31**, 1695 (1985).

10. Nosé S. A unified formulation of the constant temperature molecular dynamics methods. *J. Chem. Phys.* **81**, 511-519 (1984).

11. Plimpton S. Fast parallel algorithms for short-range molecular dynamics. *J. Comput. Phys.* **117**, 1-19 (1995).

12. Li J. AtomEye: an efficient atomistic configuration viewer. *Modell. Simul. Mater. Sci. Eng.* **11**, 173 (2003).

13. Wilcox B, Smith G. The Portevin-Le Chatelier effect in hydrogen charged nickel. *Acta Metall.* **12**, 371-376 (1964).

14. Boniszewski T, Smith G. The influence of hydrogen on the plastic deformation ductility, and fracture of nickel in tension. *Acta Metall.* **11**, 165-178 (1963).
