# Supplementary material for: Hydrogenated vacancies lock dislocations in aluminium
Source: Nat Commun. 2016 Nov 3;7:13341. doi: 10.1038/ncomms13341 (PMC5097162; doi:10.1038/ncomms13341)
Supplement: Peer Review [file ncomms13341-s5.pdf]

PEER REVIEW FILE

Reviewers' comments:

Reviewer #1 (Remarks to the Author):

The manuscript "Hydrogenated vacancies lock dislocations in aluminum" by Degang Xie et al reports on novel and very interesting results on hydrogen - dislocation interactions resolved by combining environmental TEM with mechanical stimulus inside the TEM. The experimental results are interpreted by molecular dynamic simulations. The authors have executed cyclic miniaturized compression and bending experiments on Al samples, which are prepared by Ga-FIB. The Ga induced defects are reduced by cyclic deformation to provide a starting dislocation configuration. Subsequently, the miniaturized samples are exposed to H2 and deformation is executed. The cyclic deformation reveals a hardening (which is explained by hydrogenation) followed by a softening, which is explained by unpinning from the segregated VaH defect clusters. The story sounds round, but I still see some open points which need to be discussed prior to publication:

1) The Al pillars are cut using a Ga FIB and it is well known that Ga likes to segregate in Al. Since no grain boundaries are present it is very likely that Ga segregates to the dislocations. In that case the main concern is that Ga segregating to the dislocations may change under exposure to H2. In that case Ga-hydride(s) may form. Thus, I wonder if alternative preparation routes for Al samples (e.g. Xe-FIB) would give the same results and such an artifact can be excluded.

2) The TEM experiments are performed at 300kV, while the threshold for knock-on damage is below 200kV. Can you exclude that the defect nature, e.g. the superabundant hydrogenated vacancies are not affected? I suggest to repeat some of the experiments at accelerating voltages of 120KV or lower to exclude any influence of irradiation.

3) In Figure S4 the sample H2#2 shows plasticity right from the beginning or is compressed with additional bending. In the first case the stress for dislocation activation is reduced or in the second case the data can't be interpreted.

4) Fig. S4: The small dislocation content gives already quite some statistical variation and rather than engineering stress the shear stress should be used - which of course is difficult as that would require for each dislocation to determine the glide plane. A work-around would be to estimate from the curvature of the dislocations the local stress. At the moment diagram S4b assumes that

the global stress and the local stress are identical (which can't be true due to taper of the pillars). The assumption that global stress and local stress are similar should be verified by analyzing the dislocation curvature.

5) Comment: The compression experiments are always prone to bending especially if as in this study extensive cyclic loading-unloading is performed. E.g. in Fig S2 the top part of the pillar shows no dislocation contrast due to bending, which may lead to unexpected alterations of dislocation structures. Did you think of cyclic tensile-tensile experiments?

Reviewer #2 (Remarks to the Author):

The authors write in the discussion part that "the observed stick-slip dislocation movement in our tests is consistent with the Portevin-Le Chatelier (PLC) or dynamic strain ageing (DSA) effect" (p. 11). I do not think this is true. PLC and DSA occurs when the segregating solute is diffusing at the same velocity as the gliding dislocation, so the dislocation is dragging its Cottrell atmosphere when gliding. This is therefore not consistent with the time needed for the H-Va diffusion, as estimated by the authors. Isolated H may have a high enough diffusivity at room temperatures to allow for DSA, but not H-Va complexes. The relocking of the dislocation by H-Va complexes is indeed the manifestation of static strain ageing: dislocations are at rest and the segregating complex has enough time to diffuse. I therefore think that the discussion part of the manuscript should be rewritten in consequence.

In this discussion of PLC, I do not think that the analogy between Al and Ni is meaningful. This is true that both metals have similar stacking fault energies, but Ni has stiffer elastic constants. As a consequence, the dissociation distance in Ni is at least twice the one of Al. Segregation of H or H-Va on the stacking fault ribbon (Suzuki effect) will be therefore much more important in Ni than Al.

3) H and dislocation mobility
Previous experimental studies (reference 29 for instance) have shown that H increases dislocation mobility in aluminum. Here the authors observe the opposite trend as the dislocations are pinned on unobservables obstacles assumed to be H-Va complexes. There is therefore an apparent contradiction between this work and previous studies. This needs to be discussed more thoroughly in the manuscript: why do the authors observe a different behaviour for dislocation mobility? do they have any idea of the origin of this difference? ...

4) Simulation part

Can the authors asses the validity of the empirical potential used for the atomistic simulations? They give for instance the ab-initio value (0;4 eV) for the binding energy of the H-Va complex. How the value predicted by the empirical potential compares with this value? Any other comparison between the empirical potential and ab initio or experimental data will be welcome, on migration energies for instance.

More details are also needed on the CINEB calculations of the migration energy of the H-Va complex. One can imagine several pathways resulting in such a migration. The authors should describe the different pathways they have considered so as to ensure that all "reasonable" pathways have been considered. With a migration energy of 0.4 eV for a single vacancy and 0.19 eV for a single H, I will indeed expect a migration energy close to 0.4 eV for the H-Va complex.

In the main part of the manuscript (p.9), it is not clear that the CINEB calculations rely on the empirical potential. As the authors quoted the ab initio result for the binding energy, one can think that these CINEB calculations are also relying on ab initio calculations.

In the supplementary information, when describing the MD simulations of the interaction between the dislocation and the H-Va complex (section 7), can the authors confirm that their distribution of H-Va complexes is the thermodynamic distribution corresponding to equilibrium segregation? This information is given for the interaction with H atoms (section 6), but not for H-Va complexes. In view of the discussion on static and dynamic strain ageing (point 2 above), this detail is important.

Fig. 5a:
I don't understand why the energy variation of the migrating H (green plot) is not equal to zero for the final state (reaction coordinate equal to 1).

5) Typos
p.11, first line: "further confirm" and not "further confirms"

Caption of figure S2: (c) show the abrupt jump of dislocation #2, and not #1.

Reviewer #3 (Remarks to the Author):

Review of Nature Communications manuscript NCOMMS-16-03876-T
Inbox x
Hydrogenated vacancies lock dislocations in aluminum by Xie, et al.

1. The 1000 s relocking time in the Abstract surprised this reviewer. Is this consistent with the activation energies of vacancies and hydrogen in Al?

2. Figures 1 and 2 are quite impressive in terms of the reproducibility after a 200 minute hold time. The fact that some of the dislocations moved over that time is not unusual but it is noted that one moved approximately 200 nm and others very little after 200 minutes. Were several slip systems involved?

3. In Fig. 3, the a,b,c,d is missing but assumed to be the same scheme as in fig. 2. The Fig. 3 caption states that after hydrogenation, all four dislocations immediately ceased after hydrogenation. The overall discussion of Figs. 3b and 3c is somewhat confusing at first glance. Apparently, on page 6 the black dot pinning point has locked the dislocation. This lock then disappears and the dislocation has arrested in 3c after exposure to hydrogen for 180 seconds. Why does the lock disappear? The rearrest with exposure to hydrogen in 180s compares to the 1000 seconds it takes for hydrogen-vacancy pairs (SAVs). It would be useful in a single sentence to clarify that locking of dislocations can occur by both mechanisms. Do both lead to softening by different processes or is this not obvious from the atomistic modeling? It seem counterintuitive to say that local hardening does not occur in either if it takes a larger stress to unlock dislocation? It appears that hardening and softening both exist. The extended process can be understood if dynamic strain aging leads to microviding at crack tips as you suggest by referring to Refs. 12 and 19. As a forward-looking statement, you could suggest that in-situ TEM experiments of crack-tips would be required to ascertain if such nano/micro -voiding leads to growth at the macro scale.

4. Given the historical studies of hydrogen effects on hardening of austenitic stainless steels, while you mentioned the Birnbaum and Robertson studies of the 1980s, you missed about two decades of literature associated with interstitial effects on the Portevin Le Chatelier effect. This occurred by dislocation locking in an iron-nickel-carbon system as early as 1966 by Rose and Glover in an Acta Metallurgica paper. The strain-aging process in terms of locking by carbon-vacancy pairs was reported as a serrated yielding effect of carbon-vacancy paris in Ni by Crib and Read-Hill in a 1977 Met.Trans. paper. While these are carbon based, I remember a study even earlier at lower temperatures which exhibited the P-L effect of hydrogen in Nickel. Given that, could carbon as well as hydrogen also be involved in the case of aluminum even though the solubility is nearly non-existent.? Given your focus-ion-beam deposition of carbon on the nanopillars, could any carbon have been deposited on the side walls. While solubility is nearly negligible, has this been rejected as unimportant?

Reviewers' comments:

Reviewer #1 (Remarks to the Author):

The manuscript "Hydrogenated vacancies lock dislocations in aluminum" by Degang Xie et al reports on **novel and very interesting results** on hydrogen - dislocation interactions resolved by combining environmental TEM with mechanical stimulus inside the TEM. The experimental results are interpreted by molecular dynamic simulations. The authors have executed cyclic miniaturized compression and bending experiments on Al samples, which are prepared by Ga-FIB. The Ga induced defects are reduced by cyclic deformation to provide a starting dislocation configuration. Subsequently, the miniaturized samples are exposed to H2 and deformation is executed. The cyclic deformation reveals a hardening (which is explained by hydrogenation) followed by a softening, which is explained by unpinning from the segregated VaH defect clusters. The story sounds round, but I still see some open points which need to be discussed prior to publication:

Reply: We thank the reviewer for considering our results “novel and very interesting” and the nice summary of our work.

The Al pillars are cut using a Ga FIB and it is well known that **Ga likes to segregate in Al**. Since no grain boundaries are present it is very likely that Ga segregates to the dislocations. In that case the main concern is that Ga segregating to the dislocations may change under exposure to H2. In that case **Ga-hydride(s) may form**. Thus, I wonder if alternative preparation routes for Al samples (e.g. Xe-FIB) would give the same results and such an artifact can be excluded.

Reply: Thanks for the suggestion to exclude possible effects from Ga^+. Unfortunately, Xe-FIB is not good at shaping sub-micron sized structures, due to its relative larger probe size at low milling current (e.g. 80 nm probe size at 100 pA). We do not have access to Xe ion source either. Instead of Xe, we tried to use He-FIB to fabricate pillars. But the micromachining using the light He requires a high dose, such that He bubbles form, as shown in Fig. R1.

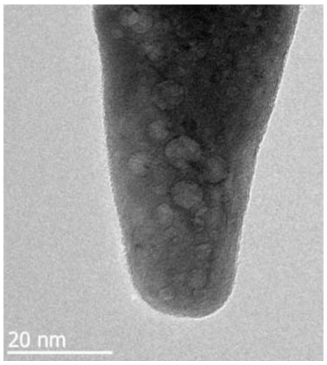

Figure R1 A gold pillar fabricated with He^+ FIB, showing abundant He bubbles due to He implantation and aggregation.

We have found an alternative way, however, to exclude the influence of Ga. Gallium hydrides are known to be usually gaseous or liquid at room temperature (see link1, link2). In vacuum, the hydride would tend to evaporate rapidly if it forms. We have therefore decided to look for Ga

using electron energy loss spectroscopy (EELS). During FIBing, Ga ions generally stop within a few tens nanometers underneath the surface. For aluminum pillars, the native oxide/metal interface should be the location where Ga would segregate to. However, our EELS mapping data shown in Fig. R2 found no appreciable Ga signal, in severely hydrogenated aluminum surface. This suggests that Ga hydride is not retained in the sample and unlikely to significantly affect dislocation behavior.

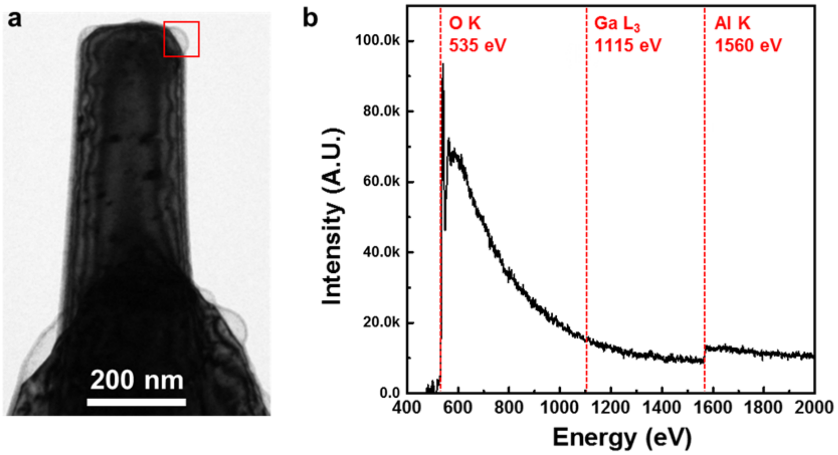

Figure R2 Chemical analysis at the hydrogenated pillar surface with electron energy loss spectroscopy (EELS). a, a pillar hydrogenated by E-beam irradiation in 2 Pa hydrogen gas for 55 min. **b**, EELS spectrum taken underneath a blister in (**a**) shows clear O-K edges and Al-K edges, but no Ga-L_3 edges, indicating that Ga segregation in insignificant in our experiments.

2) The TEM experiments are performed at 300kV, while the threshold for knock-on damage is below 200kV. Can you exclude that the defect nature, e.g. the superabundant hydrogenated vacancies are not affected? I suggest to repeat some of the experiments at accelerating voltages of 120KV or lower to **exclude any influence of irradiation.**

Reply: We agree that e-beam induced displacements can generate vacancies. But that is additional to hydrogenation-induced vacancies. In other words, irradiation only change the population of defects but not its nature. Both sources facilitate our observation of the effects of hydrogenated vacancies, in the same direction. It is difficult to achieve in situ observation without beam effects. In fact, lowering the beam energy may even cause more damage accumulation in the (hydrogen affected) depth regime we are studying: the energy transfer in each “collision” is less but the probability of collision increases.

Conventional charging techniques like chemical/electrochemical charging, plasma charging also create superabundant hydrogenated vacancies in aluminum (J Alloys Compd, 2002, 330 649), which can affect dislocation behavior like those produced by irradiation effect. In our study, we actually take advantage of the irradiation effect to generate vacancies which in turn highlight the effect of hydrogen. Therefore, in spite of the existence of irradiation effect, the general conclusions from our key findings are actually quite robust.

3) In **Figure S4 the sample H2#2** shows **plasticity right from the beginning** or is compressed with **additional bending**. In the first case the stress for dislocation activation is reduced or in the second case the data can't be interpreted.

Reply: Thanks for reading the SI carefully. These supplementary compression tests are only meant to compare stresses, as we did not calibrate the strain. We checked the original movie but didn't see more bending in H2#2 than other pillars. 'Softer' beginning may arise from slight misalignment or uneven initial contact, which affects the "strain" recorded and happens in some of the sub-micron scale tests. We have now added explanation in the SI.

4) **Fig. S4**: The small dislocation content gives already quite some statistical variation and rather than engineering stress **the shear stress should be used** - which of course is difficult as that would require for each dislocation to determine the glide plane. A work-around would be to estimate from the curvature of the dislocations the local stress. At the moment diagram S4b assumes that the global stress and the local stress are identical (which can't be true due to taper of the pillars). The **assumption that global stress and local stress are similar should be verified by analyzing the dislocation curvature.**

Reply: We totally agree that determining the shear stress is the perfect way to justifying dislocation locking. Indeed, the local stress varies inside the dislocation-rich pillars. But it is impossible to pin down the local stress in pillars with messy dislocation configurations. Again, these supplementary compression tests are only meant to show a trend (we do not rely on them to determine the shear stress). But these data inspired us to design the cyclic compression tests to get cleaner results. In these latter cases, the determination of shear stress was discussed in the paper, and the values are more reliable.

5) Comment: The compression experiments are always **prone to bending** especially if as in this study extensive cyclic loading-unloading is performed. E.g. in Fig S2 the top part of the pillar shows no dislocation contrast due to bending, which may lead to unexpected alterations of dislocation structures. Did you think of **cyclic tensile-tensile experiments**?

Reply: The contrast change for dislocation #5 in Fig. S2 is partly due to the ~2 nm lateral tip vibration, which mainly affect the contrast near the pillar top. The middle and bottom part of the pillar still have good dislocation contrast. Actually, we noticed the bending issue that sometimes did happen, an example of which was shown in Fig. S3. We tried many tests, but discarded those involving significant bending. We took many measures to minimize the bending: 1) we limit the loading cycles in each tests to avoid excessive bending. Retouching between tests was designed to relieve the bending, and the bending should be at its minimal at the beginning part of a test. 2) We always tried to use the same part of the diamond tip to execute the compression to make sure the same contact between each tests. Since multiple evidence indicate the same dislocation locking effect, the bending issue in our tests should not affect the results much.

As to the cyclic tensile experiments inside environmental TEM, it is indeed a much better choice, but it's currently unavailable to us (not yet possible in HITACHI environmental TEM with MEMS-based transducer). We are actively working with the manufacture to realize the tensile

function inside ETEM now. Anyway, based on the data collected so far, we are confident that cyclic tensile-tensile tests will yield similar results.

Reviewer #2 (Remarks to the Author):
The authors write in the discussion part that "the observed stick-slip dislocation movement in our tests is consistent with the Portevin-Le Chatelier (PLC) or dynamic strain ageing (DSA) effect" (p. 11). I do not think this is true. PLC and DSA occurs when the segregating solute is diffusing at the same velocity as the gliding dislocation, so the dislocation is dragging its Cottrell atmosphere when gliding.

Reply: The stress serration in DSA/PLC is caused by the shedding and recapturing of the Cottrell atmosphere. The timescale of the stick-slip instability provides information about the diffusivity, which is the same as the approach we take in the present paper. Averaging over a **long gliding distance** and many stick-slip cycles would give the same averaged velocity of Cottrell atmosphere and dislocation. However, our experimental setup cannot offer such long averaging distance for dislocation glide as in the bulk crystals under **constant stress**. We can only use a different setup, namely, **short glide distance** and **oscillating stress**. But both measures the same essential physical parameter, which is the diffusivity.

This is therefore not consistent with the time needed for the H-Va diffusion, as estimated by the authors. Isolated H may have a high enough diffusivity at room temperatures to allow for DSA, but not H-Va complexes. The relocking of the dislocation by H-Va complexes is indeed the manifestation of **static strain ageing**: dislocations are at rest and the segregating complex has enough time to diffuse. I therefore think that the discussion part of the manuscript should be rewritten in consequence.

Reply: We agree that our observed H-Va locking is a manifestation of static strain aging (SSA), rather than dynamic strain aging. We have rewritten the discussion/conclusion part.

In this discussion of PLC, I **do not think that the analogy between Al and Ni is meaningful**. This is true that both metals have similar stacking fault energies, but **Ni has stiffer elastic constants**. **As a consequence, the dissociation distance in Ni is at least twice the one of Al.** Segregation of H or H-Va on the stacking fault ribbon (Suzuki effect) will be therefore much more important in Ni than Al.

Reply: We agree that the analogy between Al and Ni is weak. We have decided to remove this part and defer the comparison to future discussions.

3) H and dislocation mobility
Previous experimental studies (reference 29 for instance) have shown that H increases dislocation mobility in aluminum. Here the authors observe the opposite trend as the dislocations are pinned on unobservables obstacles assumed to be H-Va complexes. There is therefore an apparent contradiction between this work and previous studies. This needs to be **discussed more thoroughly in the manuscript: why do the authors observe a different behaviour for dislocation mobility? do they have any idea of the origin of this difference?** ...
Reply: Thanks the reviewer for pointing out this. The reviewer is referring to the series of in situ TEM work published by UIUC researchers in the 1980s and 1990s. We can only speculate why their reported behavior is different, as explained in the following.

As an example, in Fig. 1 of Ref.29, the specimen used was hydrogenated to 1900 at.ppm, and then water-vapor-saturated hydrogen gas (dry hydrogen was claimed to have same impact but results weren't shown) was injected into the TEM. Their observation was made on those dislocations already at equilibrium (in "already-stopped" condition). Moreover, local stress may change due to hydrogen charging, arising from some concurrent events in the specimen such as "*crack growth to resume or to increase in speed*" and "*fresh dislocations to be generated from the crack tip*", as stated in the original literature. Also, local stress change can happen due to the following reasons: 1) chemical reaction between water vapor and sample surface. 2) the inward-diffusing hydrogen solutes. 3) External load may change due to the influence of hydrogen on the linked instrumental parts. In summary, it is difficult to ascertain that the 'constant load" condition claimed was truly constant when hydrogen was introduced. So their observation could be due to a change in loading rather than "hydrogen increased mobility".

Another consideration is that dislocation motion in FCC crystal is jerky. But their paper reported a continual-like motion, which has been greatly slowed down by the surface pinning effect. Carbon layers, a common contamination inside TEM, can be easily removed by electron irradiation in hydrogen atmosphere by mechanism of $\mathbf{C + H +} \xrightarrow{high\, kV\, e^-} \mathbf{C_x H_y\ (g.)}$, as shown in Fig. R3. The removal of surface carbon layer can weaken the surface pinning and might have caused the acceleration of dislocation velocity they reported.

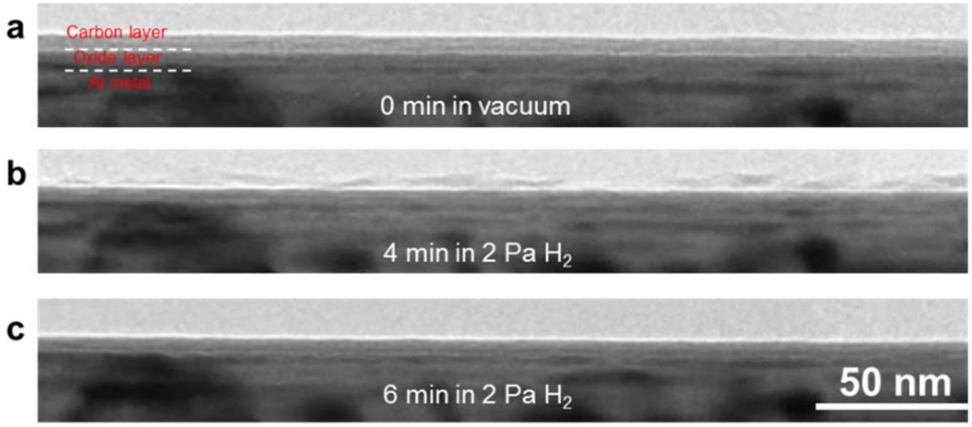

Figure R3 Removal of carbon layer by e-beam irradiation + H_2. a, the initial aluminum surface is covered with a ~5 nm carbon layer. **b**, after 4 min exposure in 2 Pa hydrogen gas, the carbon layer was partly removed, with scattered debris on surface. **c**, after 6 min exposure in 2 Pa hydrogen gas, the carbon was totally removed.

Compared to those in situ TEM works from UIUC, our experiments have the following features: 1) the loading apparatus can sense the applied force and displacement precisely. With the aid of feed-back control system, we can accurately guarantee that identical external loads were applied in vacuum tests and in hydrogen tests. 2) when charging with hydrogen, our samples are self-supported, without link to external instrumental parts. 3) we observe the bowing movements of dislocations, which are pinned at both ends.

Since 1996, the same group of researchers from UIUC actually proved that in aluminum, superabundant vacancies were always introduced irrespective of the method of hydrogen charging, and usually the vacancy concentration is comparable to or even exceeds the hydrogen concentration. But the effect of these hydrogenated vacancies on dislocation behavior has not been taken into consideration in their reports.

We have updated our manuscript to include the following sentences in the introduction part: "*But their experimental set-up could not guarantee that the applied 'constant strain' or 'constant stress' was truly constant when tens of torrs of hydrogen gas flooded the TEM chamber, as evidenced by the generation of fresh dislocations during the "constant strain" stage.*"

4) Simulation part

Can the authors asses the validity of the empirical potential used for the atomistic simulations? They give for instance the ab-initio value (0;4 eV) for the binding energy of the H-Va complex. How the value predicted by the empirical potential compares with this value? Any other comparison between the empirical potential and ab initio or experimental data will be welcome, on migration energies for instance.

Reply: To provide more details about the empirical angular-dependent potential (ADP) we used in MD simulations, here we show various properties predicted by this potential in comparison to the experiments and ab initio calculations, especially some energetics related to the point defects (H, Va and VaH), as shown in the following three Tables R1, R2 and R3. Among them, most has a relatively good match with the referenced data, but unfortunately, the binding energy of VaH is very small. For this potential, more information is shown in the papers (F. Apostol and Y. Mishin, Phys. Rev. B. 82, 144115 (2010), Y. Mishin et al, Phys. Rev. B, 59, 3393 (1999)).

Table R1 Energetics of isolated H atom in Al. ΔE_s refers to the solution energy of H in Al. T and O refer to the tetrahedral (T) interstitial and octahedral (O) interstitial sites. E_m refers to the migration barrier of one H atom from a T-site to the O-site. The units are in eV.

	ADP	ab initio
ΔE_s (T)	0.69	0.69 (ref. a)
ΔE_s (O)	0.82	0.82 (ref. a)
E_m	0.19	0.18 (ref. a)

Table R2 Energetics of Va in Al. E_v^f refers to the vacancy formation energy and E_m refers to the vacancy migration barrier. The units are in eV.

	ADP	Experiments
E_v^f	0.68	0.69 (ref. b)
E_m	0.64	0.65 (ref. c)

Table R3 Energetics of VaH. E_f (T-Va) refers to the formation energy of vacancy with H in T-site. E_f (O-Va) refers to the formation energy of vacancy with H in O-site. E_b (T-Va) refers to the

binding energy of vacancy with H in T-site. The units are in eV.

	ADP	ab initio
E_f (T-Va)	1.32	1.0 (ref. a)
E_f (O-Va)	1.36	1.14 (ref. a)
E_b (T-Va)	0.048	0.39 (ref. d)

ref. a: C. Wolverton et al, Phys. Rev. B. 69, 144109 (2004)
ref. b: H.-E. Schaefer et al, Mater. Sci. Forum 15-18, 111 (1987)
ref. c: R.W. Balluffi, J. Nucl. Mater. 67&70, 240 (1978)
ref. d: G. Lu & E. Kaxiras, Phys. Rev. Lett. 94, 155501 (2005)

However please note we did not use the ADP to perform thermodynamic calculations of H, Va and VaH partition. We simply placed VaH complexes inside the dislocation core, and study their dragging effect on the dislocation. For this particular calculation, the binding energy of H with Va to form VaH is irrelevant. The binding energy of the dislocation core with VaH is more important. Such calculation, however, would be too big to be computed by DFT. Given the very accurate matching of all other energetic parameters, we are confident the latter is calculated by ADP to good accuracy.

More details are also needed on the CINEB calculations of the migration energy of the H-Va complex. One can imagine several pathways resulting in such a migration. The authors should describe the different pathways they have considered so as to ensure that all "reasonable" pathways have been considered. With a migration energy of 0.4 eV for a single vacancy and 0.19 eV for a single H, I will indeed expect a migration energy close to 0.4 eV for the H-Va complex.
Reply: We checked different migration paths and the one we shown in the manuscript is that with the lowest energy barrier. Specifically, the equilibrium position of hydrogen atom is close to the center of tetrahedron that is formed by one vacancy and three nearest Al atoms, as shown in Fig. R4. For the diffusion of VaH complex, when the Va moves to the nearby position, the H atom has eight possible new positions around the vacancy. We calculated the migration barriers for all the eight paths and the optimal one has the value of 0.67 eV.
In addition, we also calculated the migration barrier for a single Va. The barrier (0.64 eV) is a slightly smaller than that of VaH (0.67 eV). It is very similar the case of in alpha-Fe where the hydrogenated vacancy has a higher migration barrier than that of a single vacancy (see S. Li et al, Int. J. Plast. 74, 175, 2015).

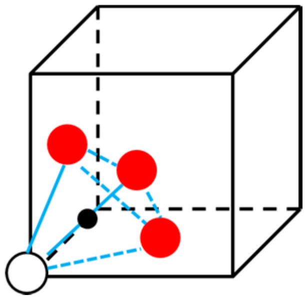

Figure R4 Schematic illustration of VaH complex migration. The open circle refers to the

vacancy. The red and black solid circles refer to Al atoms and H atom respectively.

In the main part of the manuscript (p.9), it is not clear that the CINEB calculations rely on the empirical potential. As the authors quoted the ab initio result for the binding energy, one can think that these CINEB calculations are also relying on ab initio calculations.
Reply: Thanks for pointing this out. We cited the value of binding energy from DFT calculation in order to present that Va-H complex is relatively stable in Al, like those in other metals (K. Ohsawa et al, Phys. Rev. B, 85, 094102, S. Li et al, Int. J. Plast. 74, 175, 2015). The CINEB calculations are all based on ADP potential. We revised our manuscript to avoid the misunderstanding that CINEB calculations are also relying on DFT calculations.

In the supplementary information, when describing the MD simulations of the interaction between the dislocation and the H-Va complex (section 7), can the authors confirm that their distribution of H-Va complexes is the thermodynamic distribution corresponding to equilibrium segregation? This information is given for the interaction with H atoms (section 6), but not for H-Va complexes. In view of the discussion on static and dynamic strain ageing (point 2 above), this detail is important.
Reply: Thanks for the good question. In our manuscript, we compared the pinning effect of H and VaH on dislocation motion. The H atoms were introduced around dislocation core based on the occupation probability (supplementary section 6). Actually, we also studied another kind of H configuration by distributing H atoms in a random way around the core region. We found that they take the similar pinning effect that the critical shear stress (τ_c) increased linearly with the line density of H (N_H/L), but with a lightly difference in slopes (11.7 MPa·nm for the former case and 9.1 MPa·nm for the latter case). For the distribution of VaH complexes, we first introduced vacancies randomly in the tensile region around dislocation core because the vacancies are more energy-favored in tensile region (with half-plane) than that in the compressive region. When considering energetics of VaH clusters, the H is much more preferred to bind with Va than that with Al atoms. So we added H atoms around vacancies to form VaH complexes and finally relaxed the whole systems. We gave the details in the revised supplementary information.

Fig. 5a:
I don't understand why the energy variation of the migrating H (green plot) is not equal to zero for the final state (reaction coordinate equal to 1).
Reply: We are sorry for the misunderstanding. The H migration path we showed is from one tetrahedral (T) interstitial site to the neighboring octahedral (O) site. That's why the final state is not in zero. We made it clearly in the revised manuscript.

5) Typos
p.11, first line: "further confirm" and not "further confirms"

Caption of figure S2: (c) show the abrupt jump of dislocation #2, and not #1.
Reply: Thanks for the careful scrutinize. We have corrected these typos in the new manuscript.

Reviewer #3 (Remarks to the Author):

Review of Nature Communications manuscript NCOMMS-16-03876-T
Inbox x
Hydrogenated vacancies lock dislocations in aluminum by Xie, et al.

1. The 1000 s relocking time in the Abstract surprised this reviewer. Is this consistent with the **activation energies of vacancies** and hydrogen in Al?

Reply: We were also surprised by this long relocking time. As detailed in the text, this time scale is found to be consistent with the reported measured diffusivities and activation energies, especially when compared with those of interstitial hydrogen.

In order to confirm that this relocking time is consistent with the diffusion time of Va-H complexes. We calculated the migration barrier of VaH by using CINEB method, which is around 0.67 eV (see Fig. 5a). Based on this, we can further estimate the time scale for Va-H complex to diffuse from surface to the center of the pillar (Page 9-10). The diffusion time is around 120 min, very close to the experimental observations.

2. Figures 1 and 2 are quite impressive in terms of the reproducibility after a 200 minute hold time. The fact that some of the dislocations moved over that time is not unusual but it is noted that one moved approximately 200 nm and others very little after 200 minutes. Were **several slip systems** involved?

Reply: In the text we do not exclude the possibility that these observed dislocation movements involve different slip systems. At the same time, the moving distance is also dependent on the local stress distribution, e.g., arising from pinning points, both at ends and near dislocation line.

3. In Fig. 3, the a,b,c,d is missing but assumed to be the same scheme as in fig. 2. The Fig. 3 caption states that after hydrogenation, all four dislocations immediately ceased after hydrogenation. The overall discussion of Figs. 3b and 3c is somewhat confusing at first glance. Apparently, on page 6 the **black dot pinning point** has locked the dislocation. This lock then disappears and the dislocation has arrested in 3c after exposure to hydrogen for 180 seconds. **Why does the lock disappear?** The rearrest with exposure to hydrogen in 180s compares to the 1000 seconds it takes for hydrogen-vacancy pairs (SAVs). It would be useful in a single sentence to clarify that locking of dislocations can occur by both mechanisms.

Reply: While we agree locking seems to occur by both (a) black dot, and (b) hydrogen/SAVs, we cannot control (a) which is an initial condition, while we CAN control (b) dynamically. The objective of this paper is to study (b) only, with (a) as an incidental nuisance. We believe (a) is caused by FIB Ga+ implantation, so it is an artifact with no linkage to real applications, whereas (b) does.

Do both lead to softening by different processes or is this not obvious from the atomistic

modeling**?** It seem counterintuitive to say that local hardening does not occur in either if it takes a larger stress to unlock dislocation? It appears that hardening and softening both exist.

Reply: The reviewer seems to be asking several questions: 1) what are the black dots? 2) why do the black dots disappear? 3) Do the black dots lock dislocation and cause hardening? 4) Does the release from the dots cause softening?

Origin of black dots: During the FIB process, the incident Ga^+ can displace aluminum atoms at near-surface region to form a large number of interstitial aluminum atoms, which aggregate to form interstitial disks (loops). Generally, these loops locate within a few nanometers below the surface.

While most dots disappeared during the cyclic compression process, a few of them can gradually approach the surface and suddenly escape (as shown in Fig. 2b-c). There are two reasons for the latter process: 1) these tiny loops come with **high line tension** and thus are intrinsically unstable. They tends to shrink by sucking in vacancies which flow in along the surrounding compressive stress gradient. 2) they are near-surface defects and are subjected to the **image force**, which tends to drag them towards the surface. When a dot disappears without external loading, it's difficult to discriminate whether the driving force is line tension or image force. By comparing Fig. 2b-c and Fig. 3b-c, The experimental observation is that spontaneous loss of black dots happens in both vacuum and hydrogen environment.

The black dots do pin dislocation and the loss of them can cause softening. But such hardening and softening from black dots is present without hydrogen charging. In Fig. 3, movable dislocation was found to be locked after hydrogen charging, in the absence of black dots. In Fig. 4, locked dislocations can gradually resume the movement, without losing additional black dots. The hardening and softening associated with black dots is not our focus, which is instead on "what happens to dislocations upon hydrogen charging" on top of the background events (including black dot pinning effects).

It should be noted that the 30 min hydrogenation time is equal to 1800 seconds, rather than 180 seconds quoted by the reviewer.

The extended process can be understood if dynamic strain aging leads to microviding at crack tips as you suggest by referring to Refs. 12 and 19. As a forward-looking statement, you could suggest that **in-situ TEM experiments of crack-tips** would be required to ascertain if such nano/micro - voiding leads to growth at the macro scale.

Reply: We did perform in situ observation of crack tip by bending pre-notched cantilever, and we did see voiding ahead of the fresh crack tip, as shown in Fig. R5. However, since this paper is about dislocation behavior well before void formation, we decide to remove discussions about extended processes.

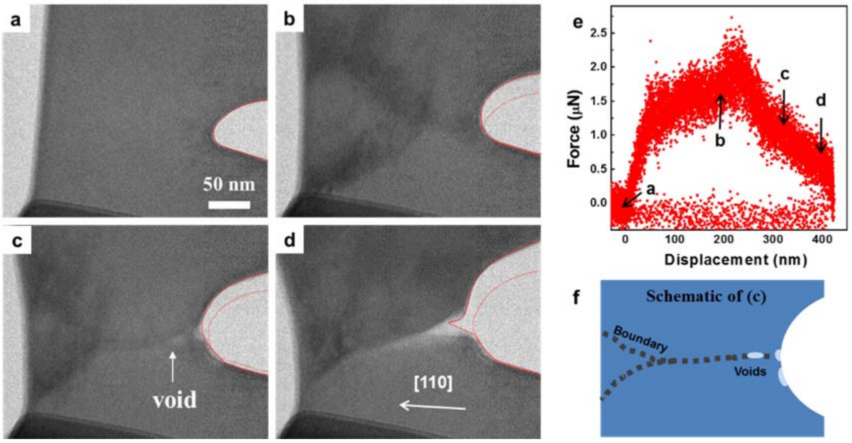

Figure R5 example of voiding during bending of a pre-notched aluminum cantilever in hydrogen environment.

4. Given the historical studies of hydrogen effects on hardening of austenitic stainless steels, while you mentioned the Birnbaum and Robertson studies of the 1980s, you missed about two decades of literature associated with **interstitial effects on the Portevin Le Chatelier effect**. This occurred by dislocation locking in an iron-nickel-carbon system as early as 1966 by Rose and Glover in an Acta Metallurgica paper. The strain-aging process in terms of locking by **carbon-vacancy pairs** was reported as a serrated yielding effect of carbon-vacancy paris in Ni by Crib and Read-Hill in a 1977 Met.Trans. paper. While these are carbon based, I remember a study even earlier at lower temperatures which exhibited the P-L effect of hydrogen in Nickel. Given that, could carbon as well as hydrogen also be involved in the case of aluminum even though the solubility is nearly non-existent.? Given your focus-ion-beam deposition of carbon on the nanopillars, **could any carbon have been deposited on the side walls**. While solubility is nearly negligible, has this been rejected as unimportant?

Reply: We have now cited those papers about hydrogen-induced PLC effect in Nickel (ref. 41, 42).

Since we deposited the carbon layer first, and then fabricated the pillars, there should be no direct carbon deposition on the side wall of these micro-milled pillars.

Carbon surface layer is a common contamination in air and in vacuum. Rather than dissolving into the pillar, we found that carbon easily disappears under the e-beam in hydrogen atmosphere, possibly due to the formation of volatile hydrocarbons, as shown in Fig. R6. The hydrogen-facilitated carbon removal can happen on the surface of any material, and even for pure carbon films. Therefore, carbon is unlikely to play any important role in our work.

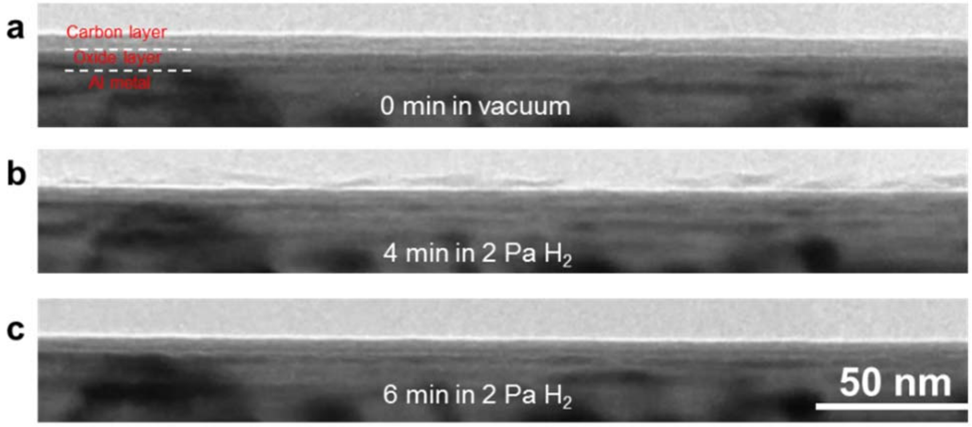

Figure R6 Removal of carbon layer by e-beam irradiation + H_2. a, the initial aluminum surface is contaminated with a ~5 nm carbon layer. **b**, after 4 min exposure in 2 Pa hydrogen gas, the carbon layer was partly removed, with scattered debris left on the surface. **c**, after 6 min exposure in 2 Pa hydrogen gas, the carbon was totally removed.

<u>Reviewers' comments:</u>

Reviewer #1 (Remarks to the Author):

The authors have given a detailed reply on all questions and concerns raised by the reviewers. I suggest to accept the manuscript for publication.

Reviewer #2 (Remarks to the Author):

Dear Editor,

The authors have satisfactorily answered to the different questions of my previous report, except the last one about the atomistic simulations with H-Va complexes. I think this last point should be addressed before the manuscript being ready for publication. I also suggest that the authors give more details on the validity of their angular dependent potential in their manuscript, and not only in the rebuttal letter.

1. MD simulations with H-Va complexes

When describing the set-up used for the molecular dynamics simulations (section 7 of supplementary information in the revised manuscript), the authors write "we first introduced vacancies randomly in the tensile region around dislocation core because the vacancies are more energy-favored in tensile region than that in the compressive region". I do not know if this is a typo in the manuscript or if this really corresponds to what has been done, but this is wrong. Vacancies in fcc metals have a negative relaxation volume. As a consequence, they prefer to lie in compressive regions, and not in tensile regions as written in the manuscript (see for instance Reference [R1]).
But the authors should actually do better than simply put randomly vacancies in the compressive regions and then add H. They should adopt the same procedure as the one used to decorate the dislocation with single vacancies (section 6 of SI): they should introduce H-Va complexes in agreement with the occupation probability of the Boltzmann distribution corresponding to the dislocation - complex binding energy. The random distribution presently used does not correspond to the static strain ageing phenomenon discussed in the manuscript. Moreover, the comparison between the hardening effect of singles vacancies with the one of H-Va complexes (Fig. 5c and S8) is meaningful only if both systems have been prepared with the same procedure, corresponding to the same physical assumptions.

2. Validity of the potential

The authors have correctly answered in their rebuttal letter to my question of the validity of their potential. I think that this details, in particular the tables R1-R3, should be included in the supplementary information. As it appears that this potential has a problem in describing the H - vacancy interaction (the binding energy is severely underestimated), this needs to be mentionned in the manuscript. The authors cannot simply write in the Method section that "This potential has a good production to various properties, such as stacking faults energy, hydrogen dissolution energy, hydrogen migration energy etc", without mentioning anything about this binding energy which is central to their study.

References:

R1 E. Clouet, "The vacancy - edge dislocation interaction in fcc metals: a comparison between atomic simulations and elasticity theory", Acta Mater. 54, 3543 (2006).

Reviewer #3 (Remarks to the Author):

Dear Authors:

While you have responded reasonably, on average, to all three reviewers, my view is that reviewers should not be put in the category of helping authors largely rewrite the paper. As rewritten it is much better but there are huge gaps of unexplained phenomena.

Reviewers' comments:

Reviewer #2 (Remarks to the Author):

1. MD simulations with H-Va complexes

When describing the set-up used for the molecular dynamics simulations (section 7 of supplementary information in the revised manuscript), the authors write "we first introduced vacancies randomly in the **tensile region** around dislocation core because the vacancies are more energy-favored in tensile region than that in the compressive region". I do not know **if this is a typo** in the manuscript or if this really corresponds to what has been done, but this is wrong. Vacancies in fcc metals have a negative relaxation volume. As a consequence, they prefer to lie in compressive regions, and not in tensile regions as written in the manuscript (see for instance Reference [R1]).

But the authors should actually do better than simply put randomly vacancies in the compressive regions and then add H. They should adopt the same procedure as the one used to decorate the dislocation with single vacancies (section 6 of SI): they **should introduce H-Va complexes in agreement with the occupation probability of the Boltzmann distribution corresponding to the dislocation - complex binding energy**. The random distribution presently used does not correspond to the static strain ageing phenomenon discussed in the manuscript. Moreover, the comparison between the hardening effect of singles vacancies with the one of H-Va complexes (Fig. 5c and S8) is meaningful only if both systems have been prepared with the same procedure, corresponding to the same physical assumptions.

References:
R1 E. Clouet, "The vacancy - edge dislocation interaction in fcc metals: a comparison between atomic simulations and elasticity theory", Acta Mater. 54, 3543 (2006).

Reply: We apologize for the typo in manuscript. The vacancies we introduced around dislocation were all in the compressive regime, consistent with that in the paper recommended by the referee.

Following referee's suggestion, we performed a new set of simulations by introducing the VaH complexes based on the Boltzmann occupation probability. Similar to that in Al-H system, we first calculated the segregation energy of VaH complex at each atomic site and further obtained $\Delta E_{\mathrm{VaH},i} (= E^{\mathrm{Edge}}_{\mathrm{VaH},i} - E^{\mathrm{Bulk}}_{\mathrm{VaH},i})$, which is the total energy difference of VaH complex in site i ($E^{\mathrm{Edge}}_{\mathrm{VaH},i}$) with reference to that in bulk site ($E^{\mathrm{Bulk}}_{\mathrm{VaH},i}$). Figure R1a shows the distribution of hydrostatic stress

of the edge dislocation and Fig. R1b shows the distribution map of $\Delta E_{\mathrm{VaH},i}$ around the dislocation core. With this map, we then introduced VaH complexes with different line densities (N_{VaH}/L) based on the Boltzmann occupation probability $p_{\mathrm{VaH},i} = e^{\Delta E_{\mathrm{VaH},i}/k_{\mathrm{B}}T} / \sum_i e^{\Delta E_{\mathrm{VaH},i}/k_{\mathrm{B}}T}$. After adding VaH complexes, we relaxed the system at 300 K for 100 ps and then performed the shear tests to study the effect of VaH complexes on the dislocation motion. The same pinning effect was observed that the critical shear stress (τ_c) shows a linear increase with the increase of VaH concentration, as shown in Fig. R2. However, in comparison to that of 75.6 MPa·nm with random distribution of VaH around dislocation core, the slope in the new model becomes even higher (101.9 MPa·nm), indicating an even stronger pinning effect. We updated the Fig. 5 in manuscript with the new model and revised the supplementary information accordingly.

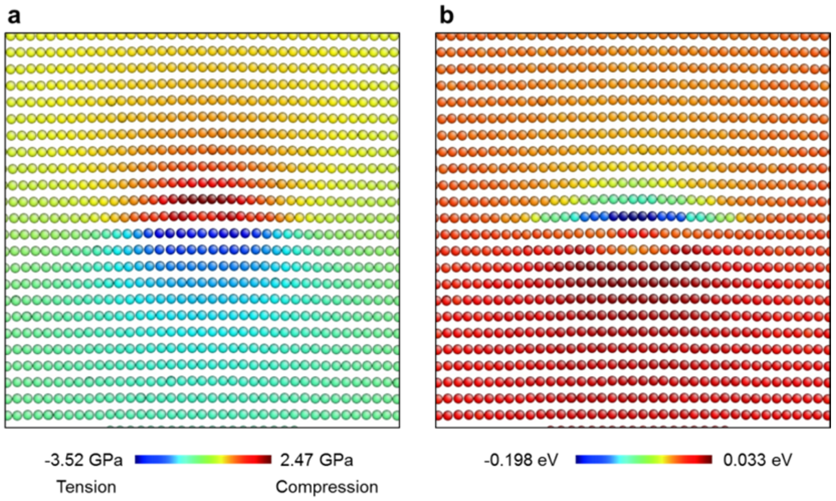

Figure R1 The distribution maps of (a) hydrostatic stress of edge dislocation in aluminum and (b) total energy difference of VaH complex ($\Delta E_{\mathrm{VaH},\,i}$).

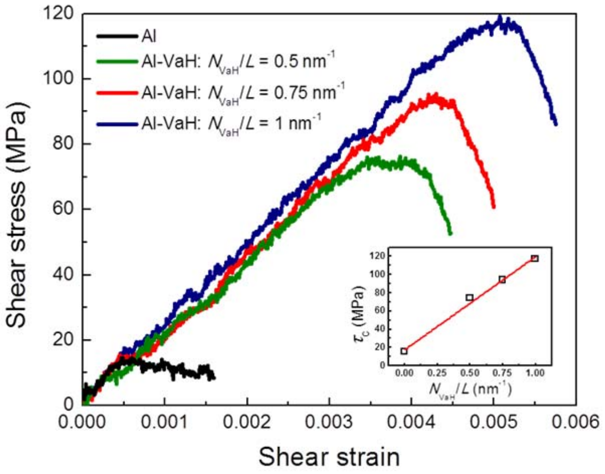

Figure R2 MD simulations of dislocation pinning effect by hydrogen-vacancy complexes. The different colored lines show the stress-strain curves at different concentrations of hydrogen-vacancy complexes. The critical shear stress (τ_c) for dislocation motion shows a linear increase with the increase of hydrogen-vacancy complex concentration (N_{VaH}/L), as shown in the inset.

2. Validity of the potential

The authors have correctly answered in their rebuttal letter to my question of the validity of their potential. I think that this details, in particular the **tables R1-R3, should be included in the supplementary information**. As it appears that this potential has a problem in describing the H - vacancy interaction (the binding energy is severely underestimated), this needs to be mentionned in the manuscript. The authors cannot simply write in the Method section that "This potential has a good production to various properties, such as stacking faults energy, hydrogen dissolution energy, hydrogen migration energy etc", without mentioning anything about this binding energy which is central to their study.

Reply: Thanks for the comment. We added more discussion about the reliability of ADP potential in Method, especially the underestimation of VaH binding energy (see highlights in the revised manuscript). We also added a new section in supplementary information (S6) to provide more information for this potential. The three tables about the energetics were also included in the new section.

<u>Reviewers' comments:</u>

Reviewer #2 (Remarks to the Author):

Dear Editor,

The authors have given clear answers to all my previous comments. I think that the manuscript can now be published.
